# Perturb-tracing enables high-content screening of multi-scale 3D genome regulators

**DOI:** 10.1038/s41592-025-02652-z

**Published:** 2025-04-10

**Authors:** Yubao Cheng, Mengwei Hu, Bing Yang, Tyler B. Jensen, Yuan Zhang, Tianqi Yang, Ruihuan Yu, Zhaoxia Ma, Jonathan S. D. Radda, Shengyan Jin, Chongzhi Zang, Siyuan Wang

**Affiliations:** 1 Department of Genetics, Yale School of Medicine, Yale University, New Haven, CT USA; 2 M.D.-Ph.D. Program, Yale University, New Haven, CT USA; 3 Department of Genome Sciences, University of Virginia, Charlottesville, VA USA; 4 Department of Biochemistry and Molecular Genetics, University of Virginia, Charlottesville, VA USA; 5 Department of Biomedical Engineering, University of Virginia, Charlottesville, VA USA; 6 UVA Comprehensive Cancer Center, University of Virginia, Charlottesville, VA USA; 7 Department of Cell Biology, Yale School of Medicine, Yale University, New Haven, CT USA; 8 Yale Combined Program in the Biological and Biomedical Sciences, Yale University, New Haven, CT USA; 9 Molecular Cell Biology, Genetics and Development Program, Yale University, New Haven, CT USA; 10 Biochemistry, Quantitative Biology, Biophysics, and Structural Biology Program, Yale University, New Haven, CT USA; 11 Yale Center for RNA Science and Medicine, Yale University School of Medicine, New Haven, CT USA; 12 Yale Liver Center, Yale University School of Medicine, New Haven, CT USA

**Keywords:** High-throughput screening, Epigenomics, Chromosomes, Nuclear organization, Fluorescence imaging

## Abstract

Three-dimensional (3D) genome organization becomes altered during development, aging and disease, but the factors regulating chromatin topology are incompletely understood and currently no technology can efficiently screen for new regulators of multi-scale chromatin organization. Here, we developed an image-based high-content screening platform (Perturb-tracing) that combines pooled CRISPR screens, a cellular barcode readout method (BARC-FISH) and chromatin tracing. We performed a loss-of-function screen in human cells, and visualized alterations to their 3D chromatin folding conformations, alongside perturbation-paired barcode readout in the same single cells. We discovered tens of new regulators of chromatin folding at different length scales, ranging from chromatin domains and compartments to chromosome territory. A subset of the regulators exhibited 3D genome effects associated with loop extrusion and A–B compartmentalization mechanisms, while others were largely unrelated to these known 3D genome mechanisms. Finally, we identified new regulators of nuclear architectures and found a functional link between chromatin compaction and nuclear shape. Altogether, our method enables scalable, high-content identification of chromatin and nuclear topology regulators that will stimulate new insights into the 3D genome.

## Abstract (web summary)

This work presents Perturb-tracing, integrating CRISPR screening with barcode readout and chromatin tracing for loss-of-function screens, enabling the identification of chromatin folding regulators at various length scales.

## Main

The spatial organization of chromatin is linked to many genomic functions and shows intriguing dynamics in a variety of biological processes and diseases^1^. Chromatin organization occurs at many levels and length scales, from local accessibility to longer-range contacts to global nuclear architecture. Studies on effectors such as histone modification enzymes or chromatin loop organizers, for example, CTCF, have bolstered our understanding of chromatin architecture, but our overarching understanding of how chromatin is regulated across length scales and in different cell types and conditions remains limited. It is a substantial challenge to systematically identify new molecular regulators of complex 3D genome architecture and form testable hypotheses about their mechanisms of action. Important 3D genome regulators have been primarily discovered by perturbing one candidate gene at a time^2–4^ or by plate-based screening focusing on relatively low-content phenotypes such as the spatial distance between one pair of genomic loci^5,6^. Recently, high-content CRISPR screens combining Perturb-seq and single-cell assay for transposase-accessible chromatin with sequencing have enabled the high-throughput discovery of candidate regulators of local chromatin accessibility^7,8^. However, we still lack scalable, broadly applicable methods to efficiently screen for regulators of higher-order 3D chromatin folding architectures, especially at the length scales of topologically associating domains (TADs, also known as contact domains), chromatin compartments and chromosome territories^9–11^.

TADs largely confine the scope of promoter–enhancer interactions^12^ and are structural units of chromatin with different DNA replication timing^13^ and mutation susceptibility^14^. TADs are further sorted into segregated A (active) and B (inactive) compartments in each chromosome territory^10^. Whole-chromosome territory compaction has been observed in X-chromosome inactivation^15^ and cellular senescence^16^. Defining the regulatory landscape and architectural basis of chromatin folding at the length scales that are relevant to each genomic feature is critical to understanding their functions and dynamics in development, aging and disease^1,17,18^. Assessing multiple length scales in parallel is technically challenging and currently only possible with single-gene perturbations.

We endeavored to develop a technique that would allow for the discovery of new factors influencing chromatin architecture as well as providing clues into which length scales and chromatin features they act on. To this end, here we present a method termed Perturb-tracing that combines the power of pooled CRISPR screening of candidate regulators with high-content readout of chromatin organization over multiple length scales. Our high-throughput, high-content genetic perturbation screen is combined with super-resolved in situ tracing of complex chromatin folding conformations and imaging of nuclear architectures across multiple length scales in human cells. A key innovation of our technology is the decoding technique we devised, termed ‘barcode amplification by rolling circle and fluorescence in situ hybridization’ (BARC-FISH), which enzymatically amplifies the barcode of each single guide RNA (sgRNA) in situ for robust decoding using fluorescence in situ hybridization (FISH), which is compatible with the multi-scale 3D chromatin mapping required to assess high-content phenotypic readouts for each barcode. After validating our method, we applied it to screen 137 candidate genes with 420 sgRNAs and imaged 30 chromatin or cellular targets per cell, generating 12,600 imaging target–perturbation combinations. The screen identified 21 top-ranking hits as new regulators of 3D genome organization at different length scales. Correlation analyses revealed regulators working in conjunction with known 3D genome regulatory mechanisms. In addition, we discovered a general link between chromatin compaction regulation and the maintenance of nuclear sphericity. Altogether, our work presented here provides a platform for the discovery and characterization of new chromatin regulators across multiple length scales and paves the way to build a global map of how 3D genomic architecture is regulated in diverse contexts.

## Results

### Perturb-tracing enables image-based screening of 3D genome regulators

**Fig. 1:**
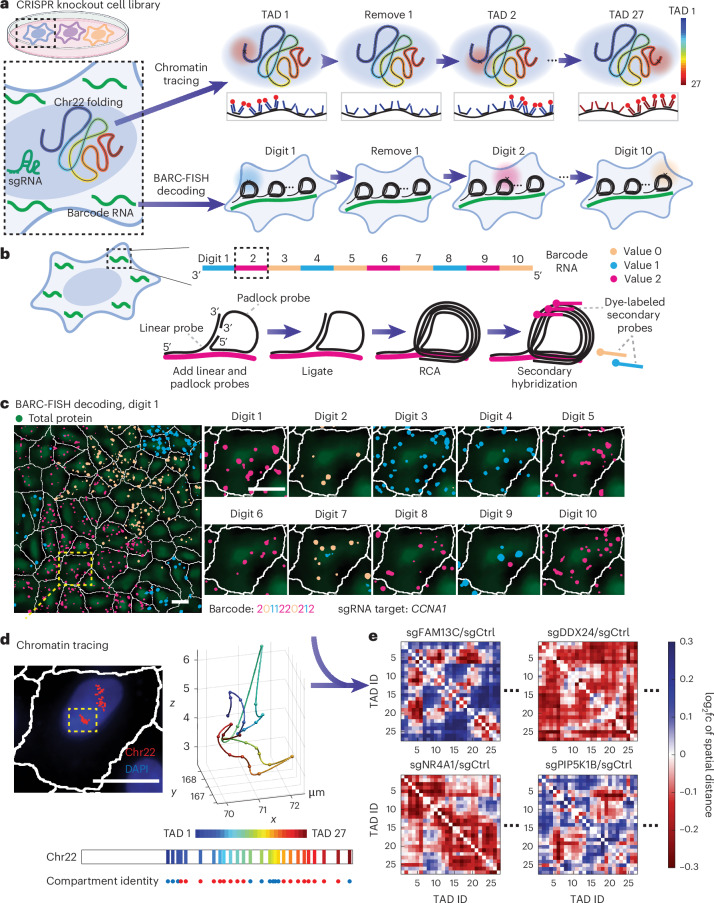
Perturb-tracing enables image-based pooled CRISPR screen of chromatin and nuclear organization regulators. **a**, Schematic of the screening approach. For chromatin tracing, all 27 TADs spanning chr22 were sequentially visualized in a multiplexed DNA FISH procedure. For BARC-FISH decoding, ten digits of the barcode were amplified and sequentially imaged. **b**, A scheme of the BARC-FISH method. The expressed barcode RNA was composed of ten ‘digits’, and each digit had one of three different values (values 0, 1 and 2, represented by orange, cyan and magenta, respectively). **c**, An example of BARC-FISH decoding from one of 17 replicate datasets. A representative field of view (left image) from the screen with BARC-FISH signals shown in orange, cyan and magenta; cell segmentation shown as white lines; and total protein stain in green. The right boxes depict magnifcations of the yellow box in the left image in ten rounds of decoding. Scale bars, 20 µm. **d**, Chromatin tracing of the yellow-boxed cell in **c**. Left, an image of the cell, with the traces of three copies of chr22 shown in red and DAPI stain shown in blue. Right, 3D chromatin trace of the chromosome in the yellow box in the left image. The 3D positions of each TAD are shown as pseudo-colored spots, connected with a smooth curve. Below, The genomic positions of TADs 1–27 on chr22, and their corresponding compartment identity (red, compartment A; blue, compartment B). Scale bar, 20 µm. **e**, Example matrices of log_2_ fold changes of inter-TAD spatial distances for selected hits from the screen.

**Extended Data Fig. 1:**
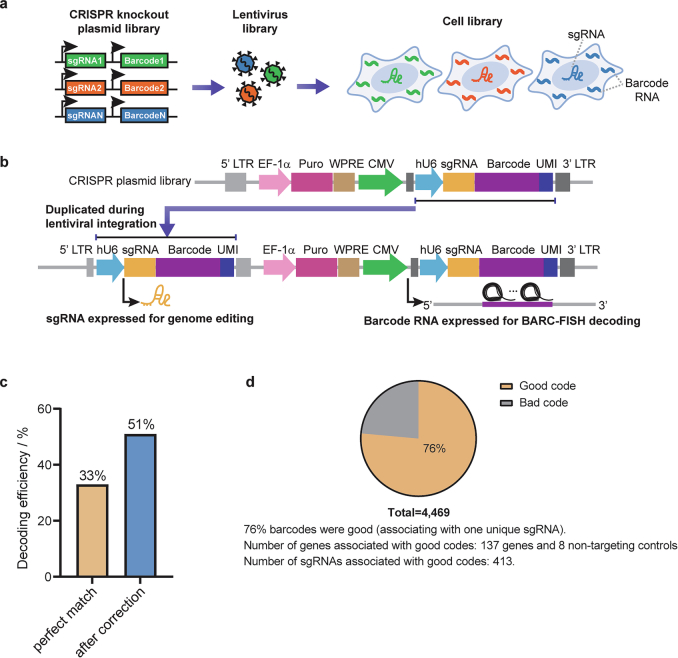
CRISPR screen library allows for sgRNA expression for genome editing and the barcode RNA expression for BARC-FISH decoding. **a**, Schematic of the construction of CRISPR screen library. A CRISPR knockout plasmid library containing sgRNA-barcode associations was constructed to generate a lentivirus library, which was transduced into human A549-Cas9 cells to produce a cell library. **b**, Design of the CRISPR screen plasmid and the lentiviral integration strategy. The sgRNA-barcode cassette was composed of human U6 promoter (hU6, blue), sgRNA (yellow), barcode (purple) and UMI (dark blue) sequences and placed within 3′ long terminal repeat (3′ LTR, dark gray), downstream of a strong RNA Pol II promoter (CMV, green). This cassette was duplicated and inserted within 5′ LTR (light gray) during lentiviral integration. Therefore, the cassette within 5′ LTR was able to express the sgRNA for genome editing, while the other copy was driven by CMV promoter to express a high level of barcode RNA for BARC-FISH decoding. Other elements on the plasmid including EF-1α promoter (pink), Puromycin resistance gene (magenta) and WPRE element (brown) were shown. **c**, BARC-FISH decoding efficiency in the CRISPR screen cell library. After BARC-FISH decoding procedure, the decoded barcodes were compared and matched to the barcodes determined by NGS. 33% of the imaged cells contained barcodes with perfect matches. After the error correction, 51% of the cells contained matched barcodes. **d**, Analysis of barcode quality determined by NGS. The sgRNA-barcode associations in the CRISPR screen cell library were determined by NGS (see Methods, “Detection of sgRNA-barcode associations in the cell library” section). In total, 4,469 barcodes were detected, among which 76% were good codes associating with one unique sgRNA. 413 sgRNAs targeting 137 genes and 8 non-targeting controls were found to be associated with these good codes. 412 of the 413 sgRNAs were observed in the image-based screen.

To systematically discover new regulators of chromatin conformation, we developed an image-based method termed Perturb-tracing to screen chromatin structures in individual knockout cells. Briefly, we used CRISPR–Cas9 technology to generate a pooled library of knockout A549 human lung cancer cells that coexpress Cas9 with a sgRNA and a unique RNA barcode (Fig. 1a and Extended Data Fig. 1a,b). The barcode RNA comprised ten regions, each encoding one of three sequences (Fig. 1b), a design inspired by a recent report^19^. Each region is analogous to a ternary digit (with values ‘0’, ‘1’ or ‘2’) in computation, and each ten-digit barcode was uniquely paired with an sgRNA in the same cell (Fig. 1a,b). We developed BARC-FISH to visually read out the barcode RNAs in single cells, and thereby identify the genetic perturbation in each cell (see below). To map the phenotypic effects of each knockout on 3D spatial organization of numerous genomic loci in single cells, we performed the highly multiplexed DNA FISH method known as chromatin tracing^20^ on chromosome 22 (chr22; Fig. 1a). Finally, we used a high-throughput computational approach to identify conserved features and systematic changes of chromatin organization caused by the same genetic perturbation in multiple cells, for every gene knockout in the pooled screen.

For BARC-FISH, we adopted a high-efficiency rolling circle amplification (RCA) strategy from an in situ sequencing method^21^. Specifically, a linear probe and a ‘padlock’ probe are hybridized to each ‘digit’ in the RNA barcode. Circularization and ligation of the padlock probe generate a template for RCA, primed by the linear probe, which in turn locally generates many copies of part of the digit sequence (Fig. 1b). For each of the ten digits, we hybridized dye-labeled secondary probes to the RCA product and observed a strong signal over background (Fig. 1b,c). To detect the value of each digit in individual cells, we sequentially applied three-color secondary probes for each digit, and imaged the fluorescence signals from the labeled RCA products over ten rounds of three-color sequential FISH imaging (Fig. 1c). After each round of imaging, the fluorescence signals were removed from the cells before the next round of sequential FISH. Finally, the fluorescence signals from all rounds of imaging were computationally converted to barcode values, and subsequently to sgRNA identities for individual cells by referring to the barcode–sgRNA associations mapped by high-throughput sequencing (Methods). With high signal-to-background ratio and an error-correcting decoding algorithm (Methods), BARC-FISH achieved robust decoding while being compatible with chromatin tracing (Fig. 1c,d and Extended Data Fig. 1c,d), allowing us to match sgRNA identities underlying genetic perturbations to 3D genome phenotypes (Fig. 1e).

**Extended Data Fig. 2:**
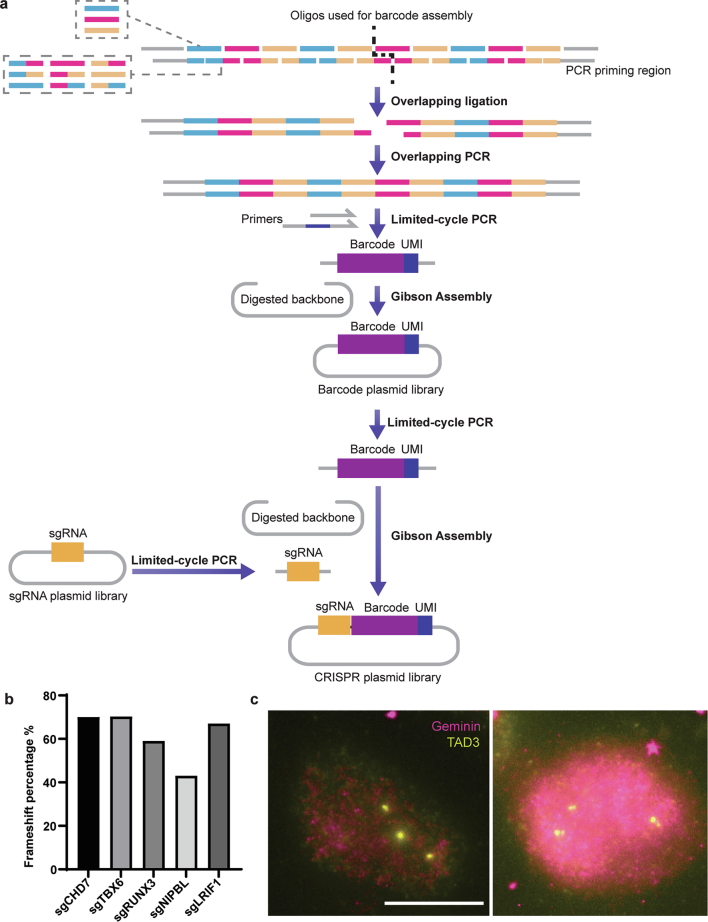
Cloning strategy of plasmid libraries, CRISPR knockout efficiency, and the Geminin-based cell cycle identification. **a**, The barcode plasmid library was assembled from individual oligos through overlapping ligation, overlapping PCR, limited-cycle PCR and Gibson Assembly (see Methods, “Barcode plasmid library construction” section). Each of the forward-strand oligos contained three alternative sequences (in the smaller gray dashed box), represented by three different colors cyan, magenta, and yellow. The overlapping oligos in the reverse strand contained 9 alternative sequences (in the larger gray dashed box). Oligos at the two ends carried PCR priming regions (straight gray lines). The barcode was divided into two halves which were subjected to overlapping ligation to form two double-stranded fragments. The two fragments were assembled by overlapping PCR to form a full-length barcode. The barcode was then amplified and added with UMI (unique molecular identifier) by limited-cycle PCR primers. The barcode-UMI fragments were inserted into a digested plasmid backbone through Gibson Assembly to construct the final barcode plasmid library. To clone the CRISPR screen plasmid library, sgRNA fragments and barcode-UMI fragments were amplified from the premade sgRNA plasmid library and barcode plasmid library respectively, through limited-cycle PCR. The sgRNA and barcode-UMI were then Gibson Assembled into a digested lentiviral plasmid backbone to generate the final CRISPR screen plasmid library (see Methods, “CRISPR screen plasmid library construction” section). The UMI was necessary for sequencing-based mapping of barcode-sgRNA associations (see Methods, “Next-generation sequencing (NGS) library preparation for mapping sgRNA-barcode associations”). **b**, Percentage of frameshift mutations of sgCHD7, sgTBX6, sgRUNX3, sgNIPBL and sgLRIF1. **c**, Two representative cells from the screen datasets were shown to demonstrate the Geminin staining strategy for G1 phase cell detection. Geminin antibody stain (magenta) is absent in a G1 phase cell (left), which showed three DNA FISH foci of TAD3 (yellow) of chr22. The S/G2 phase cell (right) is positive for Geminin stain and have six DNA FISH foci of TAD3 in three pairs, indicating replicated TAD3 DNA. Because Geminin and the yellow-green fiducial beads were imaged using the same laser channel, bead patterns were seen in both images (small, round magenta spots outside of the nuclei). Scale bar: 10 μm.

For our screen, we generated a plasmid library of 420 sgRNAs composed of 10 non-targeting control sgRNAs and 410 sgRNAs targeting 137 selected genes (a coverage of 2 to 3 sgRNAs per target gene; Supplementary Table 1). The plasmid library was cloned using a high-throughput pooled cloning strategy (Extended Data Fig. 2a and Methods). This strategy randomly paired the ten-digit barcodes with the sgRNAs and used a bottlenecking method to ensure unique mapping of each barcode to a single sgRNA. With this strategy, up to ~5,000 sgRNAs can be distinguished with the current barcoding scheme (Methods). It is possible to extend the barcode sequence to allow for more perturbations in a single screen. The 137 selected genes included ones encoding known chromatin conformation regulators such as NIPBL and CTCF as positive controls^22^, and primarily genes encoding nuclear proteins that are differentially expressed by more than fivefold upon oncogene-induced senescence^23^, given the extensive 3D genome reorganization during this process^16,18^. We generated the cell knockout library by lentiviral transduction of the plasmid library with puromycin selection, and detected 8 non-targeting sgRNA controls and 404 sgRNAs corresponding to all 137 targeted genes, suggesting an sgRNA dropout rate of 1.9% with our library construction. To estimate the knockout efficiency in our cell background, we transduced A549-Cas9 cells with selected individual sgRNA constructs and performed next-generation sequencing (NGS) on the target genomic DNA of the polyclonal cells after transduction and selection. The results showed knockout efficiencies of 43–70% for the selected sgRNAs (Extended Data Fig. 2b), close to the predicted knockout efficiencies in previously reported CRISPR editing prediction models^24,25^. This less-than-100% knockout efficiency is intrinsic to CRISPR and is expected to potentially lead to a mixture of wild-type and knockout phenotypes in subpopulations of cells carrying the same sgRNA, but we expect to still capture knockout phenotypes that are sufficiently strong in population analyses.

### Identification of regulators of multi-scale chromatin folding

For chromatin tracing, we mapped the conformation of chr22 as a model (due to its short genomic length and the lack of known structural variations on chr22 in A549 cells) at the TAD-to-chromosome length scales by pinpointing the central 100-kb regions of all 27 TADs spanning chr22 (Fig. 1d). To exclude the potential influence of cell cycle on chromatin conformation, we incorporated Geminin staining and only included G1 phase cells in our analyses (Extended Data Fig. 2c)^26^. Individual G1 phase A549-Cas9 cells contained 2–4 chr22 traces each. We analyzed 57,286 traces containing 1,407,797 3D positions from 17,304 cells.

**Fig. 2:**
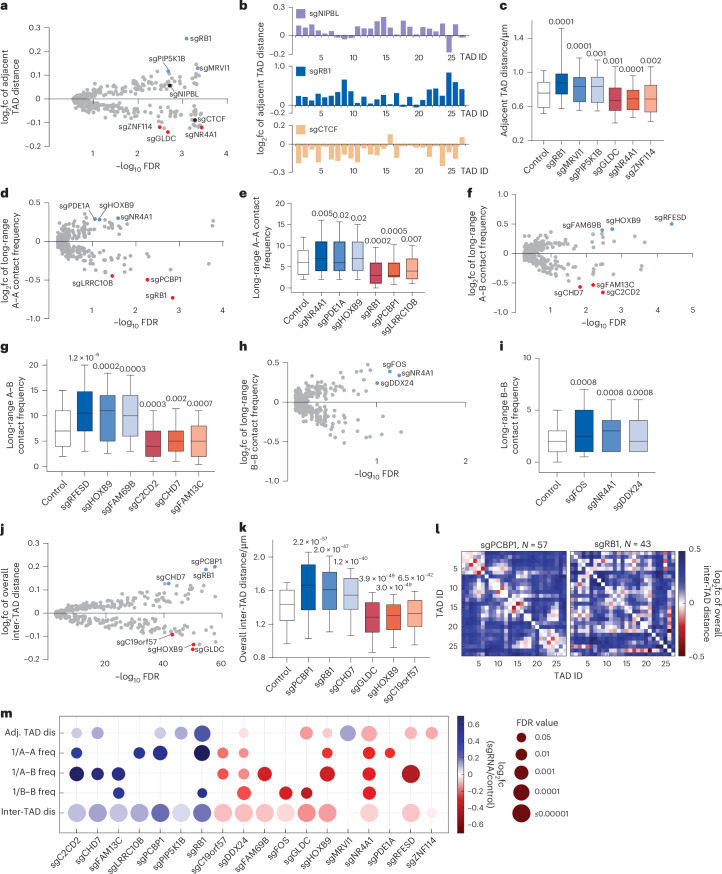
Perturb-tracing screen identified regulators of multi-scale chromatin folding. **a**, log_2_ fold change (log_2_fc) of spatial distance between adjacent TADs versus −log_10_ false discovery rate (FDR) for each perturbation. In all volcano plots, top nuclear protein hits (largest log_2_fc, FDR < 0.1) are marked: blue indicates upregulation and red indicates downregulation after knockout. **b**, log_2_fc of adjacent TAD distances across chr22 for selected hits. **c**, Spatial distances between adjacent TADs for control and selected hits. Number of traces analyzed: 57286, 43, 116, 87, 40, 129 and 42 (left to right). **d**, log_2_fc of long-range A–A contact frequency versus −log_10_ FDR for each perturbation. **e**, Long-range A–A contact frequencies for control and selected hits. Number of traces analyzed: 57286, 129, 103, 65, 43, 57 and 54 (left to right). **f**, log_2_fc of long-range A–B contact frequency versus −log_10_ FDR for each perturbation. **g**, Long-range A–B contact frequencies for control and selected hits. Number of traces analyzed: 57286, 92, 65, 50, 49, 41 and 53 (left to right). **h**, log_2_fc of long-range B–B contact frequency versus −log_10_ FDR for each perturbation. **i**, Long-range B–B contact frequencies for control and selected hits. Number of traces analyzed: 57286, 124, 129 and 213 (left to right). **j**, log_2_fc of overall inter-TAD distances versus −log_10_ FDR for each perturbation. **k**, Overall inter-TAD distances for control and selected hits. Number of traces analyzed: 57286, 57, 276, 41, 40, 65 and 61 (left to right). **l**, log_2_fc of overall inter-TAD distances in chr22 for selected hits. **m**, Fold change (circle color) and significance (circle size) of multi-scale chromatin folding phenotypes of top hits. Phenotypic changes with FDR > 0.05 are not shown. *P* values in **c** and **k** were calculated by two-sided Wilcoxon signed-rank test with FDR correction. *P* values in **e**, **g** and **i** were calculated by two-sided Wilcoxon rank-sum test with FDR correction. The boxes cover the 25th to 75th percentiles, the whiskers cover the 10th to 90th percentiles, and the lines in the middle of the boxes represent the median values.

To identify regulators of 3D genome organization, we first investigated the spatial distances between adjacent TADs on chr22. Consistent with recent imaging studies focusing on single pairs of adjacent TADs^27,28^, we found that loss of the known cohesin loader NIPBL led to a significant increase in adjacent TAD distance, whereas loss of CTCF significantly decreased adjacent TAD distance as expected^27,28^ (Fig. 2a,b). These positive control results are consistent with the opposing roles of CTCF and NIPBL in loop extrusion^2–4,27,28^ and cross-validated our screening method. Importantly, other knockout hits also altered the adjacent TAD distances, revealing new candidate regulators of chromatin organization. We observed that loss of the tumor suppressor RB1, MRVI1 and PIP5K1B increased the adjacent TAD distance, while loss of GLDC, the nuclear receptor NR4A1 and ZNF114 caused the opposite phenotype (Fig. 2a–c).

We next explored the regulation of chromatin folding conformations at the length scale of A–B compartmentalization by measuring long-range chromatin contact frequencies for each perturbation within the same cells screened above. We defined two TADs spaced less than 500 nm apart as in contact with each other, and derived the long-range contact frequency between nonadjacent TADs along the chr22 genomic map. As A–B compartment organization stems from long-range chromatin contact^10^, we further categorized the long-range contacts as contacts between compartment A regions (A–A), contacts between compartment B regions (B–B) and inter-compartmental contacts between A and B regions (A–B). We observed that loss of NR4A1, PDE1A or the homeobox transcription factor HOXB9 increased the long-range A–A contact frequency, while loss of RB1, PCBP1 or LRRC10B showed the opposite phenotype (Fig. 2d,e). Loss of RFESD, HOXB9 or FAM69B increased the frequency of inter-compartmental A–B contacts, while loss of C2CD2, the chromatin remodeler CHD7 or FAM13C decreased A–B contacts (Fig. 2f,g). Finally, loss of the AP-1 transcription factor subunit FOS, NR4A1 or the helicase DDX24 increased the long-range B–B contact frequency (Fig. 2h,i).

Finally, we integrated all pairs of inter-TAD distances to measure and compare the overall compactness of the chr22 chromosome territory. The average fold changes of all 351 inter-TAD distances among the 27 TADs were calculated. We found that knocking out *PCBP1*, *RB1* or *CHD7* decompacted chr22 (Fig. 2j–l), while *GLDC*, *HOXB9* or *BRME1* (*C19orf57)* knockout resulted in chr22 compaction (Fig. 2j,k). Intriguingly, RB1 is known to promote the formation of senescence-associated heterochromatin foci^29^, a highly compacted whole-chromosome conformation. Our results show that *RB1* knockout decompacts chr22 across multiple scales, including increased adjacent TAD distance (Fig. 2a–c), reduced long-range A–A contact frequency (Fig. 2d,e) and overall decompacted chromosome territory (Fig. 2j–l). The high-content readouts of our screen are, therefore, in agreement with this prior association but, importantly, provide new insights into the scales at which RB1 and the other hits impact chromatin organization.

We noticed that several chromatin folding regulators identified above, such as RB1, NR4A1, GLDC, HOXB9, PCBP1 and CHD7, were called as top hits in more than one architectural category. This observation led us to ask if chromatin folding regulators in general tend to affect multiple chromatin architectures across different length scales. To this end, we quantified the regulatory effects of each top hit on all five architectural features analyzed above (adjacent TAD distances, A–A, B–B and A–B interactions, and whole-chromosome compaction). The results showed that most top hits significantly affect chromatin folding in more than one architectural category (Fig. 2m). In general, the top hits can be classified into chromatin compactors that reduce inter-loci distances and increase contact frequencies, and chromatin decompactors with the opposite function, although the extents of the regulatory effects often differ between categories for a given regulator (Fig. 2m). These observations indicate 3D genome regulators often have a multi-scale effect.

### Correlation analyses link new regulators to known 3D genome mechanisms

**Fig. 3:**
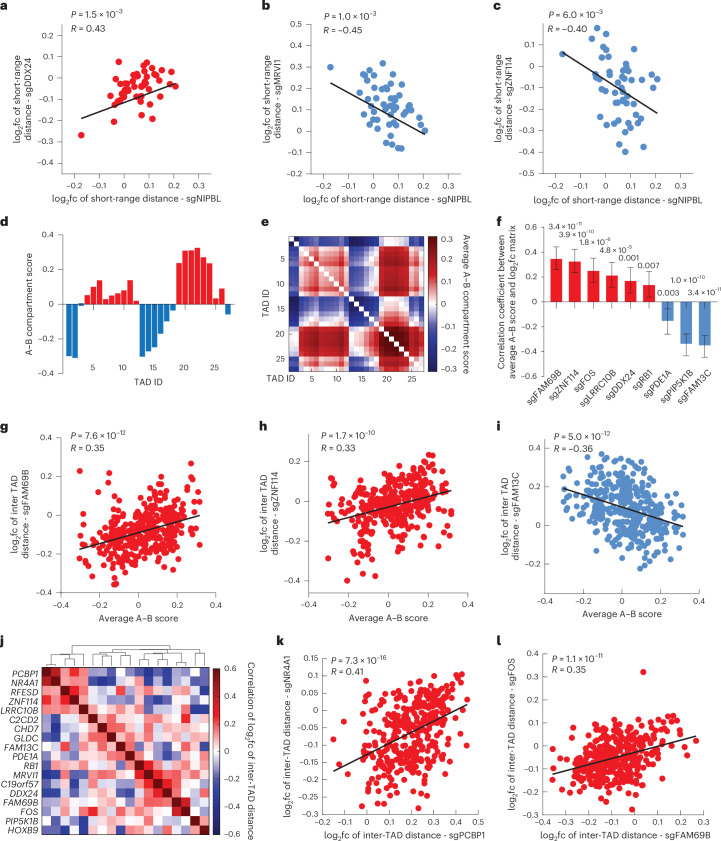
Characterization of the regulators of multi-scale chromatin folding. **a**–**c**, Correlation of log_2_fc of short-range inter-TAD distances (defined as spatial distances between genomic regions that are less than 3 Mb apart) between sgNIPBL and sgDDX24 (**a**), sgMRVI1 (**b**) or sgZNF114 (**c**). **d**, A–B compartment score profile of chr22. **e**, Matrix of average A–B compartment scores of pairs of TADs. **f**, Top hits with 3D genome effects (log_2_fc of inter-TAD distance upon knockout) significantly correlated with the A–B compartment score matrix. Number of traces analyzed: 50, 42, 124, 54, 213, 43, 103, 87 and 53 (left to right). Correlations were derived from 351 data points. Data are presented as correlation coefficients with 95% confidence intervals as error bars. **g**–**i**, Correlation between the average A–B compartment score of the TADs and the log_2_fc of inter-TAD distances upon FAM69B (**g**), ZNF114 (**h**) or FAM13C (**i**) knockout. **j**, Hierarchical clustering of correlation coefficients between the log_2_fc matrices of inter-TAD distances for top hits. **k**,**l**, Correlation of the log_2_fc of inter-TAD distances between sgPCBP1 and sgNR4A1 (**k**) or between sgFAM69B and sgFOS (**l**). *P* values in **a**–**c**, **f**–**i** and **k**–**l** were calculated by two-sided *t*-test. FDR corrections were applied to **f**.

The high-content nature of our screen offers whole-distance matrices for individual perturbations, allowing for correlation analyses between the 3D genome regulatory effects of the new regulators and those of previously identified mechanisms. Exploiting this capacity, we first quantified the correlations between each new regulator and NIPBL in controlling short-range (<3 Mb) chromatin distances to detect potential mechanistic associations with the loop extrusion mechanism^30^. We found DDX24, MRVI1 and ZNF114 showed significant correlations with NIPBL (Fig. 3a–c), suggesting at least partial interactions or involvement with the loop extrusion mechanism, although the involvement may be indirect.

We next asked whether the regulatory effects of the top hits are associated with or mediated by the A–B compartmentalization scheme. We converted the one-dimensional A–B compartment score profile to a 2D matrix by calculating the average A–B compartment score between pairs of TADs (Fig. 3d,e), and measured the correlations between this average A–B score matrix and the log_2_ fold change matrices of inter-TAD distances for individual top hits. Of the 18 top hits, 9 showed significant correlations in this analysis, among which *FAM69B*, *ZNF114*, *FOS*, *LRRC10B*, *DDX24* and *RB1* knockout led to distance changes positively correlated with the average A–B score, whereas *PDE1A*, *PIP5K1B* and *FAM13C* knockout showed distance changes negatively correlated with the average A–B score (Fig. 3f–i). These results indicate that a subset of our identified top hits at least partially interact with or are modulated by the A–B compartmentalization mechanism. We further calculated a correlation coefficient matrix of the 18 top hits and used hierarchical clustering to group top hits that are more correlated with each other in their 3D genome effects. We observed that PCBP1, NR4A1, RFESD, ZNF114 and LRRC10B formed a cluster with correlated 3D genome effects (Fig. 3j,k). In addition, some other pairs of hits also showed significant correlations, such as FOS and FAM69B (Fig. 3j,l). These correlations suggest potential co-regulatory mechanisms of the 3D genome.

### Individual validations of selected top hits

**Fig. 4:**
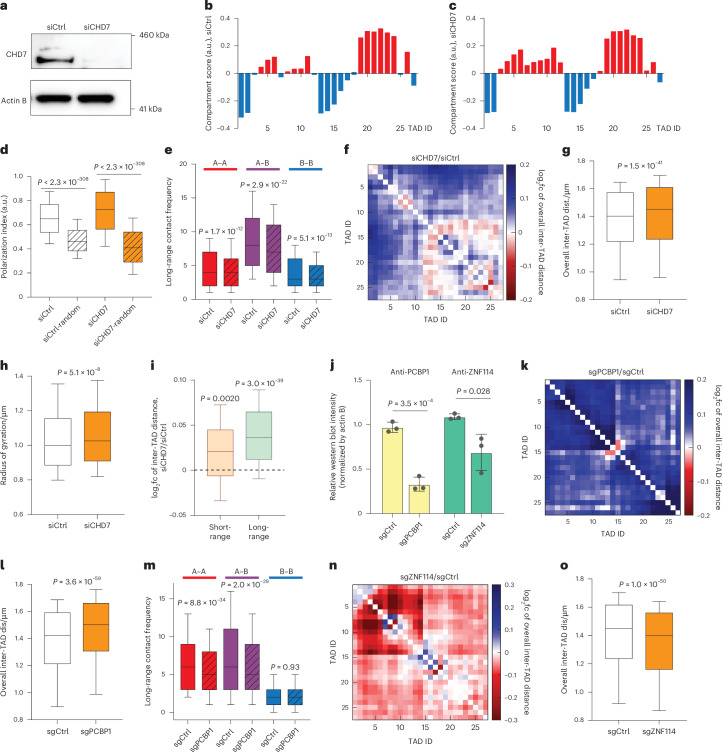
Individual validations of top hits. **a**, Western blot of siCtrl-treated and siCHD7-treated A549-Cas9 nuclear extracts. The experiment was repeated five times with similar results. **b**, A–B compartment profile of chr22 in siCtrl cells. **c**, A–B compartment profile of chr22 in siCHD7 cells. **d**, Polarization indices of chr22 A–B compartments of siCtrl (white) and siCHD7 (orange). Shadowed boxes show the polarization indices from randomized controls, where the compartment identities of TADs are scrambled. **e**, Compartmental contact frequencies of siCtrl and siCHD7 (shadowed) among A compartment regions (red), between A and B compartment regions (purple) and among B compartment regions (blue). **f**, log_2_fc of inter-TAD distance of siCHD7 compared to siCtrl. **g**, Overall inter-TAD distance of siCtrl and siCHD7. **h**, Radii of gyration of siCtrl and siCHD7. **i**, log_2_fc of short-range and long-range inter-TAD distances between siCHD7 and siCtrl. **j**, Relative blot intensities of PCBP1 and ZNF114 western blot bands (normalized by loading control actin B bands). Data are presented as mean values ± s.d. Statistics are derived from three biological replicates. **k**, log_2_fc of inter-TAD distance of sgPCBP1 compared to sgCtrl. **l**, Overall inter-TAD distance of sgCtrl and sgPCBP1. **m**, Compartmental contact frequencies of sgCtrl and sgPCBP1 (shadowed) among A compartment regions (red), between A and B compartment regions (purple) and among B compartment regions (blue). **n**, log_2_fc of inter-TAD distance of sgZNF114 compared to sgCtrl. **o**, Overall inter-TAD distance of sgCtrl and sgZNF114. *P* values in **d**, **e**, **h** and **m** were calculated by two-sided Wilcoxon rank-sum test. *P* values in **g**, **i**, **l** and **o** were calculated by two-sided Wilcoxon signed-rank test. *P* value in **j** was calculated by a two-sided, two-sample *t*-test. The boxes cover the 25th to 75th percentiles, the whiskers cover the 10th to 90th percentiles, and the lines in the middle of the boxes represent the median values. Number of traces analyzed: 3,181 (siCtrl) and 3,545 (siCHD7) for **d** and **h**; 3,558 (siCtrl) and 4,134 (siCHD7) for **e**–**g** and **i**; 4,627 (sgCtrl) and 3,543 (sgPCBP1) for **k**–**m**; 4,602 (sgCtrl) and 3,954 (sgZNF114) for **n** and **o**. a.u., arbitrary units. Source data

As we examined our hits, CHD7 drew our attention because it regulated chromatin conformation on different scales in opposing ways. CHD7, or chromodomain helicase DNA binding protein 7, is a chromatin remodeler known to promote local chromatin openness and is associated with CHARGE syndrome^31–33^. The results of our screen showed that *CHD7* knockout significantly reduced long-range A–B contact frequencies (Fig. 2f,g) and resulted in decompaction of the whole chr22 territory (Fig. 2j,k). To validate this large-scale chromatin compaction function of CHD7 using an orthogonal method, we performed knockdown of *CHD7* using small interfering RNA (siRNA) in the same A549-Cas9 cell line. Western blot analysis confirmed that the CHD7 protein level was reduced by >90% (Fig. 4a). Chromatin tracing of chr22 in the *CHD7* knockdown (siCHD7) and control (siCtrl) cells showed that while the compartment identities of TADs were largely identical between siCtrl and siCHD7 (Fig. 4b,c), and the A–B compartments were spatially positioned in a conventional, polarized manner^20^ in both cases (Fig. 4d), A–A, B–B and A–B contacts were less frequent in siCHD7 compared to siCtrl cells (Fig. 4e). *CHD7* knockdown also caused overall chromosome territory decompaction, represented by a global increase in inter-TAD distances (Fig. 4f,g) and larger radii of gyration of the whole-chromosome traces (Fig. 4h). The spatial regulatory effect of CHD7 appeared stronger at long range (>3 Mb) compared to short range (<3 Mb; Fig. 4i). Altogether, these results agree with the phenotypes we observed for CHD7 in our knockout screen, confirming that this factor facilitates chromatin compaction especially at long range.

**Extended Data Fig. 3:**
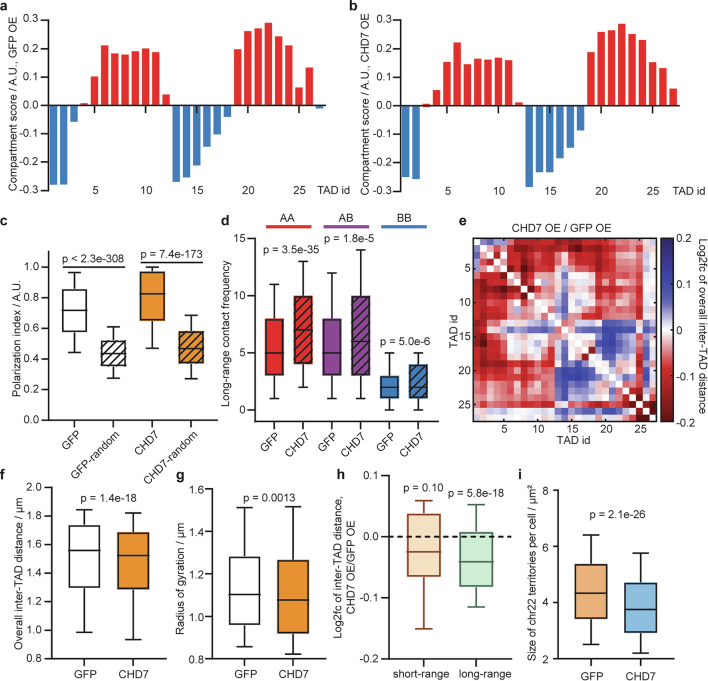
Validation of CHD7 perturbation phenotypes using overexpression. **a**, A-B compartment profile of chr22 in A549-Cas9 cells with GFP overexpression. **b**, A-B compartment profile of chr22 in A549-Cas9 cells with CHD7 overexpression. **c**, Polarization indices of cells with GFP (white) and CHD7 (orange) overexpression and the corresponding randomized controls (shadowed). Number of traces analyzed: 2,701 (GFP and GFP-random) and 1,015 (CHD7 and CHD7-random). **d**, Compartmental contact frequencies of cells with GFP of CHD7 (shadowed) overexpression in A compartments (red), across A and B compartments (purple) and in B compartments (blue). Number of traces analyzed: 3,157 (GFP OE) and 1,174 (CHD7 OE). **e**, Log2 fold change of inter-TAD distance of CHD7 overexpression compared to GFP overexpression. Number of traces analyzed: 3,157 (GFP OE) and 1,174 (CHD7 OE). **f**, Overall inter-TAD distance of chr22 in cells with GFP and CHD7 overexpression. Number of traces analyzed: 3,157 (GFP OE) and 1,174 (CHD7 OE). **g**, Radii of gyration of chr22 in cells with GFP and CHD7 overexpression. Number of traces analyzed: 2,701 (GFP OE) and 1,015 (CHD7 OE). **h**, Log2 fold change of short-range and long-range inter-TAD distances between CHD7 and GFP overexpression. Number of traces analyzed: 3,157 (GFP OE) and 1,174 (CHD7 OE). **i**, Area of chr22 territories per cell measured by whole chromosome paint in cells with GFP and CHD7 overexpression. Number of cells analyzed: 1,715 (GFP OE) and 1,538 (CHD7 OE). P values in **c**, **d**, **g** and **i** were calculated by two-sided Wilcoxon rank sum test. P values in **f** and **h** were calculated by two-sided Wilcoxon signed rank test. The boxes cover the 25^th^ to 75^th^ percentiles, the whiskers cover the 10^th^ to 90^th^ percentiles, and the lines in the middle of the boxes represent the median values.

To further validate the regulatory effect of CHD7 on chromatin organization, we overexpressed CHD7 in the A549-Cas9 cell line. In comparison to control GFP overexpression using the same vector, CHD7 overexpression significantly compacted chromatin and promoted chromatin contacts across larger length scales. While the A–B compartment identities of TADs and the polarized arrangement of A–B compartments largely remained unchanged (Extended Data Fig. 3a–c), CHD7 overexpression led to significantly higher contact frequencies between A–A, B–B and A–B compartment regions (Extended Data Fig. 3d), and significantly decreased global inter-TAD distances and radii of gyration (Extended Data Fig. 3e–g), indicating overall chromatin compaction. Similarly to the *CHD7* knockdown scenario, the compaction effect upon CHD7 overexpression was more significant at long range (Extended Data Fig. 3h). In addition to the chromatin tracing analyses above, we also used whole-chromosome paint (Methods) as an alternative readout method of chromosome territory compaction. Whole-chromosome paint of chr22 showed smaller chromosome territories upon CHD7 overexpression in comparison to control (Extended Data Fig. 3i). Together, these data further validate the findings from our screen, and show that CHD7 specifically promotes long-range chromatin compaction and contact.

**Extended Data Fig. 4:**
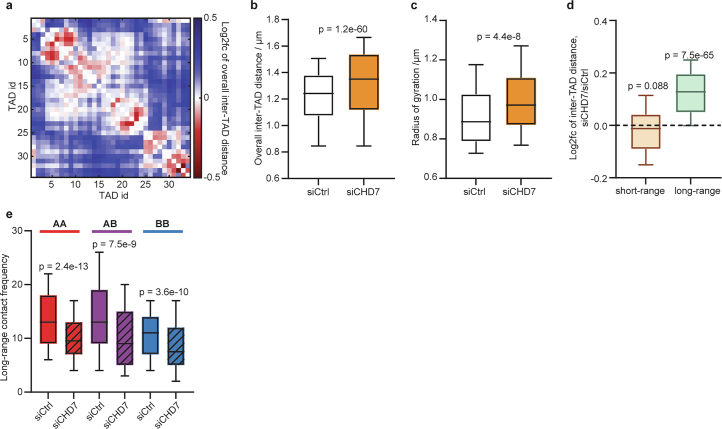
Validation of CHD7 perturbation phenotypes in a different cell background and genomic context. **a**, Log2 fold change matrix of overall inter-TAD distance of chr21 between siCHD7 and siCtrl in hTERT RPE-1 cells. Number of traces analyzed: 904 (siCtrl) and 210 (siCHD7). **b**, Overall inter-TAD distance of chr21 in siCtrl and siCHD7 cells. Number of traces analyzed: 904 (siCtrl) and 210 (siCHD7). **c**, Radii of gyration of chr21 in siCtrl and siCHD7 cells. Number of traces analyzed: 840 (siCtrl) and 178 (siCHD7). **d**, Log2 fold change of short-range and long-range inter-TAD distances between siCHD7 and siCtrl. Number of traces analyzed: 904 (siCtrl) and 210 (siCHD7). **e**, Compartmental contact frequencies in A compartments (red), across A and B compartments (purple) and in B compartments (blue) of chr21 in siCtrl and siCHD7 cells. Number of traces analyzed: 904 (siCtrl) and 210 (siCHD7). P values in **b** and **d** were calculated by two-sided Wilcoxon signed rank test. P values in **c** and **e** were calculated by two-sided Wilcoxon rank sum test. The boxes cover the 25^th^ to 75^th^ percentiles, the whiskers cover the 10^th^ to 90^th^ percentiles, and the lines in the middle of the boxes represent the median values.

**Extended Data Fig. 5:**
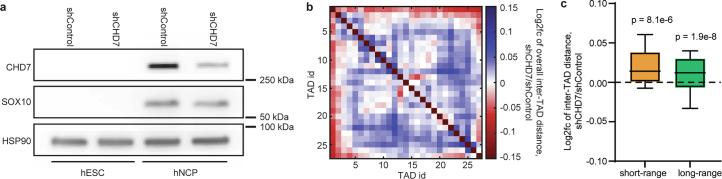
Validation of CHD7’s long range chromatin compaction function in neural crest cells. **a**, Western blot of shControl- and shCHD7-transduced human embryonic stem cells (hESC) and human neural crest progenitors (hNCP). Top: anti-CHD7 antibody; middle: anti-SOX10 antibody; bottom: anti-HSP90 antibody. CHD7 increased upon neural crest induction, and reduced in shCHD7 hNCP cells compared to shControl. Sox10, the neural crest marker, was expressed at similar levels in shControl and shCHD7 hNCP cells. HSP90 is a loading control. The experiment was repeated twice independently with similar results. **b**, Log2 fold change of overall inter-TAD distance of chr22 between shCHD7 and shControl hNCP cells. Number of traces analyzed: 4,657 (shControl) and 2,796 (shCHD7). **c**, Log2 fold change of short-range and long-range inter-TAD distances of chr22 between shCHD7 and shContrl hNCP cells. Number of traces analyzed: 4,657 (shControl) and 2,796 (shCHD7). P values were calculated by two-sided Wilcoxon signed rank test. The boxes cover the 25^th^ to 75^th^ percentiles, the whiskers cover the 10^th^ to 90^th^ percentiles, and the lines in the middle of the boxes represent the median values. Source data

To investigate if the chromatin compaction function of CHD7 applies to a different cell background and different genomic context, we performed knockdown of *CHD7* using siRNA in human RPE-1 cells and conducted chromatin tracing on chromosome 21 (chr21). *CHD7* knockdown caused an overall increase in inter-TAD distance (Extended Data Fig. 4a,b) and an increase in the radius of gyration of chr21 territory (Extended Data Fig. 4c), which were mainly contributed by long-range chromatin decompaction (Extended Data Fig. 4d). *CHD7* knockdown also led to decreased contact frequency between A–A, A–B and B–B interactions (Extended Data Fig. 4e). As CHARGE syndrome is known as a neural crest disease and the multiple organs affected by the syndrome are derived from neural crest progenitor cells in early development^34^, we further tested whether siCHD7 affects long-range chromatin compaction in neural crest progenitor cells differentiated from cultured human embryonic stem cells. Western blots confirmed the neural crest cell identity and effective knockdown of *CHD7* (Extended Data Fig. 5a). Chromatin tracing analyses confirmed that siCHD7 in neural crest cells caused long-range chromatin decompaction (Extended Data Fig. 5b,c). Together, these results indicate that the chromatin compaction function of CHD7 exists in distinct genomic contexts and cell lines, including the cell type of origin of CHARGE syndrome.

**Extended Data Fig. 6:**
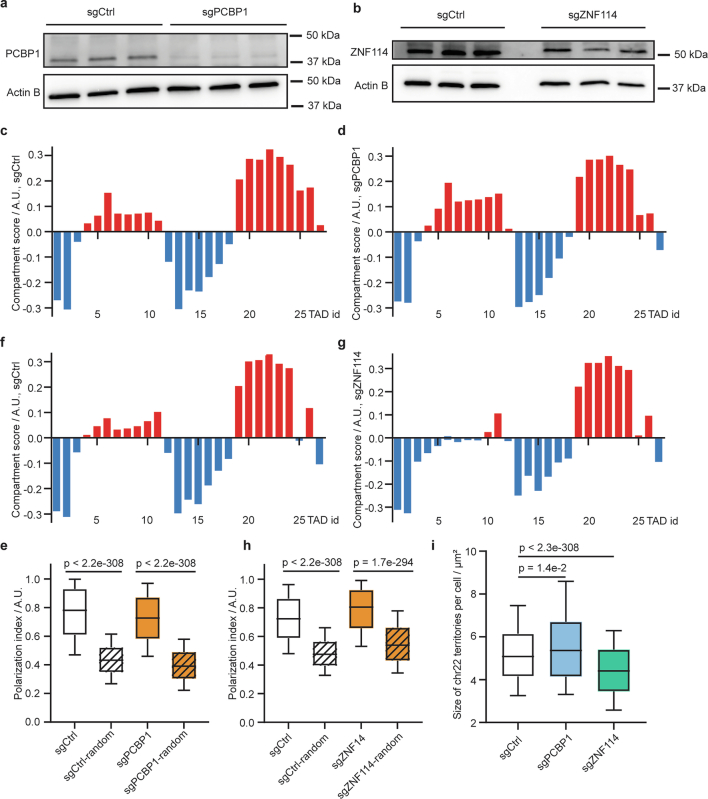
Additional PCBP1 and ZNF114 perturbation results. **a**, Western blot of sgCtrl and sgPCBP1 A549-Cas9 nuclear extracts. Top: anti-PCBP1 antibody; bottom: anti-Actin B antibody. The experiment was repeated twice independently with similar results. **b**, Western blot of sgCtrl and sgZNF114 A549-Cas9 nuclear extracts. Top: anti-ZNF114 antibody; bottom: anti-Actin B antibody. The experiment was repeated twice independently with similar results. **c-d**, A-B compartment profile of chr22 in sgCtrl (**c**) and sgPCBP1 (**d**) A549-Cas9 cells measured in parallel. **e**, Polarization indices of chr22 A-B compartments of sgCtrl (white) and sgPCBP1 (orange). Shadowed boxes show the polarization indices from randomized controls, where the compartment identities of TADs are scrambled. **f-g**, A-B compartment profile of chr22 in sgCtrl (**f**) and sgZNF114 (**g**) A549-Cas9 cells measured in parallel. **h**, Polarization indices of chr22 A-B compartments of sgCtrl (white) and sgZNF114 (orange). Shadowed boxes show the polarization indices from randomized controls, where the compartment identities of TADs are scrambled. **i**, Area of chr22 territories per cell measured by whole chromosome paint in sgCtrl, sgPCBP1 and sgZNF114 A549-Cas9 cells. Number of cells analyzed: 855 (sgCtrl), 320 (sgPCBP1) and 609 (sgZNF114). P values in **e**, **h** and **i** were calculated by two-sided Wilcoxon rank sum test. The boxes cover the 25^th^ to 75^th^ percentiles, the whiskers cover the 10^th^ to 90^th^ percentiles, and the lines in the middle of the boxes represent the median values. Source data

We further individually validated two more top hits, PCBP1 and ZNF114, using CRISPR knockout in A549-Cas9 cells. Western blots confirmed the effectiveness of the knockouts (Fig. 4j and Extended Data Fig. 6a,b). Consistent with the primary screen results, chromatin tracing targeting chr22 showed that *PCBP1* knockout led to decompaction of chromatin folding at multiple scales, including a global increase in inter-TAD distances and decrease in long-range A–A and A–B contact frequencies (Fig. 4k–m), while *ZNF114* knockout led to a global compaction of chromatin folding (Fig. 4n,o). Whole-chromosome paint of chr22 consistently showed larger chromosome territories upon *PCBP1* knockout and smaller chromosome territories upon *ZNF114* knockout (Extended Data Fig. 6i). Neither of the perturbations significantly affected the compartment identities of TADs or the polarized organization of the A–B compartments (Extended Data Fig. 6c–h). Altogether, our individual validations of CHD7, PCBP1 and ZNF114 as new regulators of 3D genome folding using various perturbation methods, cell backgrounds, genomic contexts and readout modalities support the effectiveness of the primary Perturb-tracing screen.

### Identification of nuclear morphology regulators

**Fig. 5:**
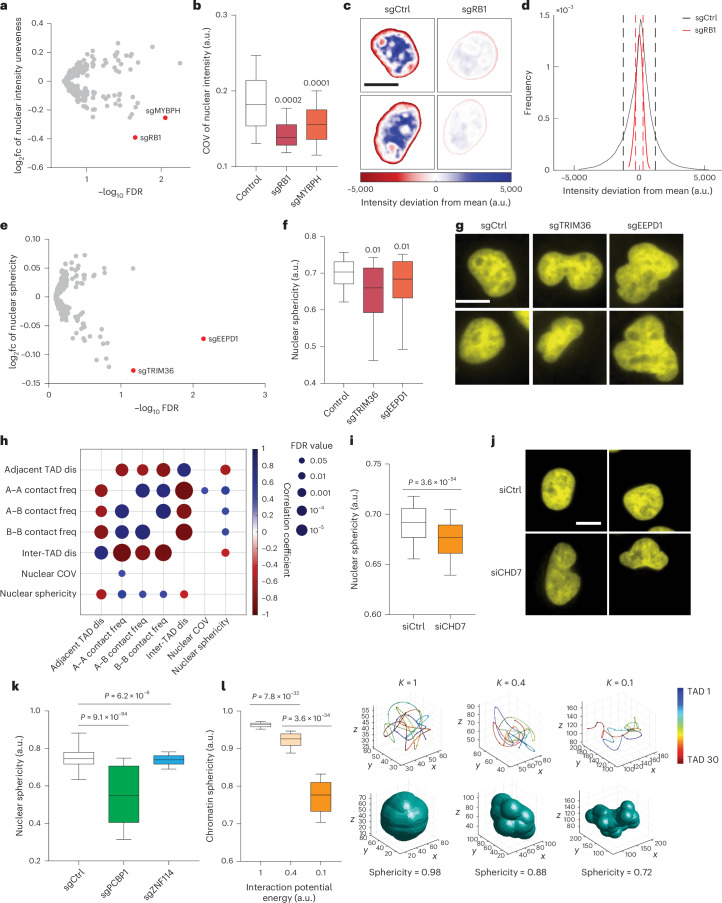
Perturb-tracing screen identified hits that regulate the morphological properties of nuclei. **a**, log_2_fc of nuclear intensity unevenness (measured as coefficient of variation (COV) of nuclear voxel intensities) versus −log_10_ FDR. **b**, Nuclear intensity unevenness of control and selected hits. Number of cells analyzed: 17304, 12 and 36 (left to right). **c**, Heat map of nuclear intensity deviation from mean intensity of representative nuclei from non-targeting control (left column) and hit sgRB1 (right column). Scale bar, 10 μm. **d**, Voxel intensity distribution of all nuclei from non-targeting control (black curve) and hit sgRB1 (red curve). Dashed lines indicate the standard deviations of the distributions. **e**, log_2_fc of nuclear sphericity versus −log_10_ FDR. **f**, Nuclear sphericity of control and selected hits. Number of cells analyzed: 17304, 14 and 64 (left to right). **g**, Representative nuclei images of non-targeting control (left column) and selected hits that regulate nuclear sphericity, TRIM36 (middle column) and EEPD1 (right column). Scale bar, 10 μm. **h**, Correlation coefficients (bubble color) and significance of correlations (bubble size) between pairs of 3D genome/nucleome features calculated using all top hits. **i**, Nuclear sphericity values of siCtrl and siCHD7 A549-Cas9 Cells. Number of cells analyzed: 1,156 (siCtrl) and 1,412 (siCHD7). **j**, Representative DAPI images of siCtrl and siCHD7 A549-Cas9 cells. **k**, Nuclear sphericity values of sgCtrl, sgPCBP1 and sgZNF114 A549-Cas9 Cells. Number of cells analyzed: 2,474 (sgCtrl), 1,395 (sgPCBP1) and 1,367 (sgZNF114). **l**, Simulated chromatin polymer folding conformations and the corresponding bounding envelop sphericity values at different chromatin self-interaction energies (*K* = 1, 0.4 or 0.1). Lower energy corresponds to weaker chromatin interaction. *N* = 100 simulated conformations for each energy. *P* values in **b**, **f**, **i**, **k** and **l** were calculated by two-sided Wilcoxon rank-sum test. *P* values in **h** were calculated by two-sided *t*-tests. FDR correction was applied to **b**, **f** and **h**. The boxes cover the 25th to 75th percentiles, the whiskers cover the 10th to 90th percentiles, and the lines in the middle of the boxes represent the median values.

Nuclear DAPI staining bears surprisingly rich information regarding the nuclear organization that can be used to distinguish cell states^35^. Chromatin is distributed unevenly in the cell nucleus, and the ‘texture’ of nuclear staining pattern is often used as a diagnostic marker of cancer^36^. Moreover, cells with abnormal, non-spherically shaped nuclei are often seen in cancer and aging and may indicate genome instability^37–39^. By analyzing changes in DAPI staining caused by each perturbation in our screen, we found that knockout of *RB1* or *MYBPH* reduced the unevenness of nuclear DAPI staining, generating patterns with more homogeneous intensity within each nucleus (Fig. 5a–d), whereas a more heterogeneous intensity pattern with chromatin condensates is often associated with cancer^36^. In addition, knocking out *TRIM36* and *EEPD1* decreased the sphericity of the nuclei and led to multi-lobed nuclei shapes (Fig. 5e–g).

To test if the nuclear phenotypes are fully orthogonal or at least partially linked to the chromatin folding phenotypes analyzed before, we calculated the correlations among the changes of different chromatin folding and nuclear organization features upon perturbations of the top hits. As expected, all chromatin folding features (adjacent TAD distances, A–A, B–B and A–B contact frequencies and inter-TAD distances) were significantly correlated with each other (Fig. 5h), consistent with the common muti-scale and multifaceted effects of the chromatin folding regulators (Fig. 2m). Interestingly, nuclear sphericity also showed significant correlations with chromatin folding features across length scales and categories (Fig. 5h). In general, chromatin compaction (decreased inter-loci distance or increased contact frequency) is associated with a more spherical nuclear shape (Fig. 5h). To validate this observation, we measured and compared the nuclear sphericity values of siCHD7 and control A549-Cas9 cells. Indeed, *CHD7* knockdown led to a less spherical and more multi-lobed nuclear shape (Fig. 5i,j). Furthermore, sgPCBP1 and sgZNF114 cells showed decreased and increased nuclear sphericity, respectively, in comparison to controls (Fig. 5k), consistent with the general trend, even though the extent of the increase in nuclear sphericity in sgZNF114 cells was minimal, likely due to the already highly spherical shapes of the control nuclei. These observations are consistent with previous reports that increasing euchromatin or decreasing heterochromatin (which likely causes large-scale chromatin decompaction) leads to less spherical and more multi-lobed nuclear morphologies^40–44^, further supporting the effectiveness of the Perturb-tracing method. To interpret this result, we performed simulation of a minimal chromatin polymer model. We showed that reduced monomer–monomer interaction strength (less chromatin compaction/interaction) can lead to a less globular chromatin folding organization, and a bounding envelope surrounding the polymer will in turn adopt a more multi-lobed shape with lower sphericity (Fig. 5l). Overall, our experimental results suggest a general link between chromatin compaction and nuclear shape, and our simulation supports a model that chromatin compaction mediates nuclear morphology.

**Extended Data Fig. 7:**
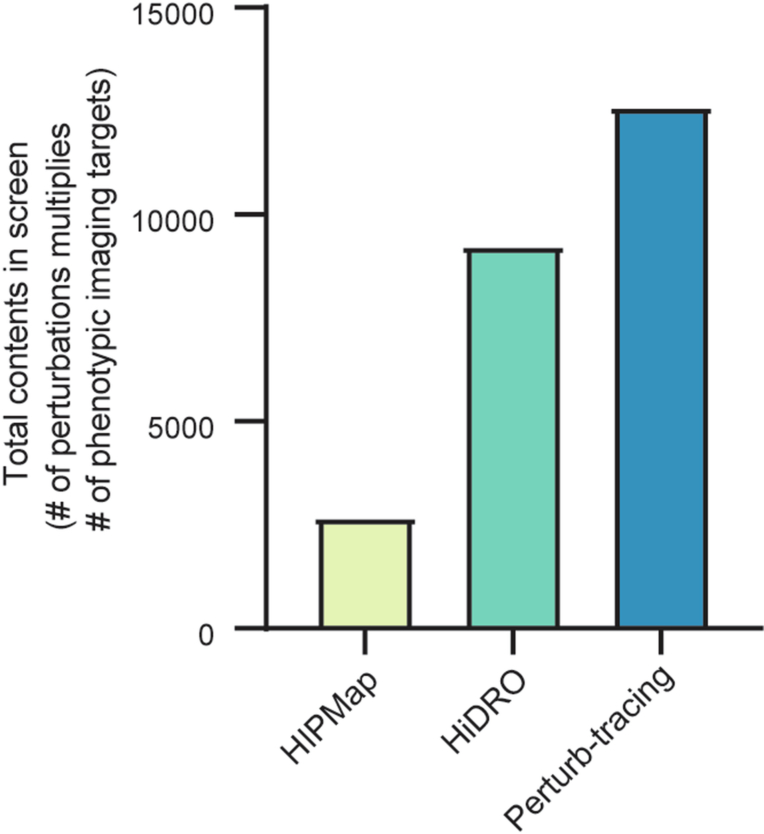
Total contents in different screen methods. Perturb-tracing: 420 gRNAs multiply 30 phenotypic imaging targets including 27 TADs, DAPI, total protein, and Geminin stains.

In summary, our image-based Perturb-tracing screen using pooled CRISPR perturbations, BARC-FISH and multi-scale chromatin profiling allowed us to systematically and simultaneously profile the effect of hundreds of candidate genes on many aspects of spatial genome organization. In this work, we identified 21 top candidate regulators of chromatin/nuclear organization from our screen of 137 selected genes (Supplementary Table 2). Our work represents a high-content screen among recent image-based 3D genome regulator screens, with ~13,000 imaging target–perturbation combinations (Extended Data Fig. 7).

## Discussion

Here, we report the development of a high-throughput, high-content, image-based genetic screening platform termed Perturb-tracing and demonstrate its ability to systematically identify regulators of 3D genome folding architectures from short-range to long-range and global nuclear organization. The Perturb-tracing platform integrates a pooled CRISPR knockout screen comprising hundreds of candidate genes with chromatin tracing and the development of a cellular barcoding and in situ decoding technique termed BARC-FISH. Other FISH-based in situ genotyping techniques have previously been reported in genetic screens but are limited to bacterial applications and/or not integrated with 3D genomics^19,45,46^. The BARC-FISH barcode design has the capacity to simultaneously assess libraries of up to ~5,000 sgRNAs in pooled format with the current experimental design (see the Methods for an explanation) and can be further extended in the future with longer barcodes and/or with more sequence varieties at each digit of the barcode. Importantly, the phenotypic readout of this screening platform is information rich, allowing for categorization of a gene of interest based on its effects on many different length scales and aspects of chromatin organization. The rich information and high-content nature of the screen also readily enables detection of associations between new regulators and known 3D genome organizers, and discovery of potentially functional linkages and co-regulation mechanisms between different 3D genome and nucleome features.

We note some cytoplasmic proteins included in our screen showed 3D genome phenotypes. For example, *AQP3* knockout led to a decrease in adjacent TAD distances (Supplementary Table 13). We hypothesize that these cytoplasmic proteins may indirectly regulate the 3D genome through cellular metabolism, signaling and/or mechanotransduction. Indeed, a recent plate-based RNA interference (RNAi) screen discovered many cytoplasmic signaling proteins that regulate the spatial distance between adjacent TADs^6^. The cytoplasmic proteins might not be direct regulators but could act through downstream effector molecules.

Whereas a recent plate-based RNAi screen focused on the druggable genome and many target genes do not encode nuclear proteins^6^, our screen focused on mostly nuclear protein-coding genes with significant expression changes in cellular senescence to increase the chance of discovery of direct regulators of the 3D genome. We intentionally retained transcription factors among our candidates, as some classic 3D genome regulators, such as CTCF, were originally identified as transcription factors. We suspect that many previously identified master transcription factors in fact are novel 3D genome regulators, and may regulate transcription through their 3D genome function. A caveat of both RNAi and CRISPR loss-of-function screens is that the depletion of target proteins may take several cell cycles, and during this time the cells may undergo major changes that affect the 3D genome through secondary effects. Thus, neither the previous plate-based screen nor the current version of Perturb-tracing can readily distinguish direct versus indirect regulators of the 3D genome. A CRISPR activation screen^47^ that rapidly upregulates target proteins in combination with Perturb-tracing may address this issue.

In our initial screen of 137 candidate genes with multiple sgRNAs per gene, we identified 21 previously unknown or incompletely known regulators of 3D genome and nuclear organization features across multiple length scales. Our seemingly high hit rate (21/137) is due to our screen observing not one but many phenotypes, which essentially combined many screens into one. In comparison to a recent plate-based screen on largely one phenotype, which reported a hit rate of 10%^6^, our effective hit rate per phenotype is lower, which reflects the high stringency of our hit calling to ensure high confidence of our top hits. Indeed, all three new regulators we tested in individual validations showed consistent results as in the primary screen. Our high stringency hit calling and the use of relatively early CRISPR knockout gRNA designs^48^ may cause false negatives, which can be addressed in future works by using more sophisticated CRISPR designs and lower hit calling stringency.

Overall, Perturb-tracing enables mapping of the chromatin organization ‘regulatome’ at scale, which will deepen our understanding of the regulatory landscape of the genome and of the functions of genome architectures.

## Methods

### Barcode and probe sequence design

#### Design of BARC-FISH barcodes, linear and padlock probes

We first designed 60 barcode-targeting regions of linear and padlock probes using 46 published 20-nucleotide-long sequences that only contain A, T and C^49^ and 14 newly designed sequences. The 14 extra sequences were generated using a previously introduced orthogonal 25-nucleotide oligonucleotide dataset^50^, by trimming off 5 consecutive nucleotides on the 5′ or 3′ end of the 25-nucleotide sequences, and only sequences that met the following three criteria were retained: (1) the sequence only contains A, T and C; (2) the sequence does not contain four or more consecutive C’s; and (3) the percentage of C is between 40% and 45%. The newly designed 20-nucleotide sequences were pooled with the 46 published sequences, and all these 20-nucleotide sequences were BLASTed among themselves to ensure they were orthogonal to each other^51^. Finally, we BLASTed all possible pairs of concatenated 20-nucleotide sequences separated by a G spacer against human genome and transcriptome to ensure they were orthogonal to endogenous nucleotide sequences^51^. We then grouped the 60 sequences into 30 pairs, and reverse-complemented the sequences to generate the DNA segments of barcode digits, each of which is 41-nucleotide long (two 20-nucleotide sequences with a 1-nucleotide spacer). We adopted the backbone sequences of linear and padlock probes from a previous work^21^ to generate the full-length linear and padlock probe sequences, with the following modification: The backbone of padlock probes of all sequences with a value of 0 for all barcode digits carried a 20-nucleotide secondary probe targeting region. For value 1 and 2 sequences of the barcode digits, the 20-nucleotide region of the padlock probe that binds to the barcode RNA serves as the secondary probe targeting region. Twenty-seven secondary probe sequences were previously reported^49,52^. The other three secondary probe sequences were designed with a previously introduced procedure^53^. The linear and padlock probes, and secondary probes labeled with ATTO 565, Alexa Fluor 647 and Alexa Fluor 750 dyes were individually ordered from Integrated DNA Technologies (IDT). A list of DNA barcode segment sequences and their corresponding linear, padlock and secondary probe sequences are included in Supplementary Table 3.

#### BARC-FISH helper probe design

To improve the hybridization efficiency of the linear and padlock probes, we designed helper probes targeting the flanking regions of the barcode RNA, which could help open potential secondary structures and linearize the RNA molecules. To design the helper probes, we adapted the algorithm of a published probe designing tool ProbeDealer^54^. Briefly, three RNA regions were used as the input targeting regions for ProbeDealer: an 888-nucleotide region upstream of the protospacer, a 108-nucleotide region between the protospacer and barcode, and a 310-nucleotide region downstream of a unique molecular identifier (UMI) region (for the region layout, refer to the sections on plasmid library construction below). The probes were generated with the following constraints: The probe length was 30 nucleotides, with at least 1-nucleotide spacing between adjacent probes; the allowed melting temperature range was 66–100 °C; the allowed GC content range was 30–90%; the melting temperature of the internal secondary structure of each probe was no greater than 76 °C; the melting temperature of cross-hybridization regions among the probes was no greater than 72 °C; and the probes containing consecutive repeats of more than five identical nucleotides were excluded. The probe sequences were further BLASTed against the human genome and transcriptome to ensure the hybridization specificity^51^. Probes with alignments to either the human genome or the human transcriptome were excluded from the selection. In total, 36 helper probes were generated and ordered through IDT. The sequences of the helper probes are listed in Supplementary Table 4.

#### Chromatin tracing primary probes

We used the primary probes of human chr21 and chr22 from a previous publication^20^ with slight modifications on secondary probe-binding regions. Specifically, the probes consisted of the following segments from the 5′ to 3′: (1) a 20-nucleotide priming region at the 5′ end; (2) a secondary probe-binding region of 17–19 nucleotides; (3) a 30-nucleotide primary binding region complementary to genomic DNA; (4) a same secondary probe-binding region of 17–19 nucleotides; and (5) a 20-nucleotide priming region at the 3′ end. The secondary probe-binding regions were generated by trimming the 5′ or 3′ ends of previously published 30-nucleotide secondary probe-binding regions^20^, so that the melting temperature of the trimmed secondary probe-binding regions became 50–57 °C.

### Barcode plasmid library construction

The DNA sequences of the ten-digit barcodes each comprised ten 41-nucleotide digit segments, with a nucleotide ‘C’ separating adjacent digits. Each digit contained one of three different 41-nucleotide sequences, representing the three different values (0, 1, 2) of the ternary digit. The values from the ten digits were randomly assembled. Therefore, theoretically 3^10^ or 59,049 possible unique combinatorial barcodes could be generated. To assemble the barcode library, we used a pooled barcode cloning strategy (Extended Data Fig. 2a) modified from approaches previously described^19,46^: First, the ten digits were divided into two fragments. Overlapping oligonucleotides encoding and bridging digits 1–5 were mixed to a final concentration of 100 nM each, and the mixture was subjected to phosphorylation and ligation. Oligonucleotides encoding digits 6–10 were treated in the same way in parallel. Then, the two five-digit fragments were gel purified and assembled into full-length barcodes by overlapping PCR. Next, the full-length barcodes were amplified by limited-cycle PCR. In this step, each barcode molecule was paired with a UMI), a 20-base pair (bp) random sequence, at the 3′ end for later NGS, and overhangs at both ends for Gibson Assembly. The PCR products were gel purified and then assembled into a plasmid backbone digested with MluI (New England Biolabs, R3198S) and SpeI (New England Biolabs, R3133S), through Gibson Assembly (New England Biolabs, E2621L). The backbone plasmid was modified from plasmid LentiGuide-Puro (Addgene, 52963), with the addition of a CMV promoter, an EGFP gene and two restriction sites, MluI and SpeI. Lastly, the Gibson reaction products were purified by isopropanol precipitation and electroporated into Endura electrocompetent cells (Lucigen, 60242-2). The transformed bacteria were spread onto LB agar plates containing 100 µg ml^−1^ ampicillin and incubated overnight at 37 °C. On the next day, ~70 million colony-forming units grown on the plates were collected and subjected to Maxi Prep (Qiagen, 12362) to extract the barcode plasmid library. The barcode plasmid library was then used to generate the CRISPR screen plasmid library. All oligonucleotides and primers were ordered from IDT. The oligonucleotide and primer sequences for constructing the barcode plasmid library are provided in Supplementary Table 5. The modified backbone sequence with annotations is included in Supplementary Data 1. An example barcode plasmid sequence with annotations is included in Supplementary Data 2.

### sgRNA plasmid library construction

To construct the sgRNA plasmid library, we selected protospacer sequences from a previous publication^48^. The sgRNA fragments were amplified from a CustomArray oligonucleotide pool via limited-cycle PCR. The backbone was digested with FastDigest Esp3I (Thermo Fisher Scientific, FD0454) together with alkaline phosphatase (Thermo Fisher Scientific, EF0654) overnight at 37 °C. The backbone plasmid was modified from plasmid LentiGuide-Puro with the addition of a CMV promoter and an EGFP gene. The sgRNA fragments and digested backbone were gel purified and assembled through Gibson Assembly. The Gibson reaction products were purified by isopropanol precipitation and the electroporated into Endura electrocompetent cells. The cells were plated onto LB agar plates containing 100 µg ml^−1^ ampicillin and incubated at 37 °C overnight. About 2.5 million colony-forming units were collected and inoculated into 200 ml LB liquid medium containing 100 µg ml^−1^ ampicillin, and grown overnight to amplify the sgRNA plasmid library. The sgRNA plasmid library was maxi-prepped from the overnight culture and used for the construction of the CRISPR screen plasmid library. All primers were ordered from IDT. The protospacer sequences, ordered CustomArray oligonucleotide sequences and primer sequences are provided in Supplementary Table 6. The modified backbone sequence with annotations is included in Supplementary Data 3. An example sgRNA plasmid sequence with annotations is available in Supplementary Data 4.

### CRISPR screen plasmid library construction

We adopted the cloning strategies from previous publications^19,55^ to construct the CRISPR screen plasmid library (Extended Data Fig. 2a). In brief, sgRNA, barcode and backbone were assembled through Gibson Assembly. The barcode fragments were amplified by limited-cycle PCR from the premade barcode plasmid library. Similarly, the sgRNA fragments were PCR amplified from the premade sgRNA plasmid library. The backbone plasmid was modified from plasmid CROP-seq-Guide-Puro (Addgene, 86708), with an addition of a CMV promoter and removal of the gRNA scaffold. The modified plasmid was restriction digested with FastDigest Esp3I overnight at 37 °C. The restriction reaction product was treated with alkaline phosphatase to remove the phosphate groups from the linearized backbone. The DNA fragments of sgRNA, barcode and backbone were gel purified and mixed for Gibson Assembly. The Gibson reaction products were purified by isopropanol precipitation and electroporated into Endura electrocompetent cells. Electroporated cells were grown overnight at 37 °C on LB agar plates containing 100 µg ml^−1^ ampicillin. To restrict the number of barcodes paired with each sgRNA and allow unique projection from each barcode to a single sgRNA, we used a bottlenecking strategy: Only ~4,000–5,000 bacterial colonies were collected from the plates so that the number of bacterial colonies is one order of magnitude lower than the total number of barcodes (3^10^ = 59,049), ensuring the vast majority of barcodes in the retained pool each occur only once, and are each uniquely paired with a gRNA. The number of collected bacterial colonies also reflects the maximum number of different gRNAs our strategy could distinguish with the current 10-trit barcode design, which is one order of magnitude lower than the total number of barcodes. Collected colonies were cultured in 200 ml LB liquid medium overnight at 37 °C with shaking at 225 rpm. The CRISPR screen plasmid library was then extracted and purified by Maxi Prep. In our modified CROP-seq backbone, the U6-gRNA-barcode sequences are inserted within the 3′ long terminal repeat (LTR) and will thus be duplicated into the 5′ LTR during lentiviral integration. After the duplication, both copies of U6 promoters can drive the expression of gRNAs for genome editing. Meanwhile, a CMV promoter can drive the transcription of a longer RNA spanning the U6-gRNA-barcode sequence in the 3′ LTR with the barcode sequences for BARC-FISH detection. This design ensures that the duplicated U6-driven gRNA expression from the 5′ LTR is not subjected to interference by the CMV promoter^56^. All primer sequences are provided in Supplementary Table 7. The modified backbone sequence with annotations is included in Supplementary Data 5. An example CRISPR screen plasmid sequence with annotations is available in Supplementary Data 6.

### Lentivirus production and cell line construction

#### Lentivirus production

The HEK-293FT cells (Thermo Fisher Scientific, R70007) were cultured to be 70–90% confluent at the point of transfection to produce lentiviruses. The Cas9 plasmid lentiCas9-Blast (Addgene, 52962), the GFP overexpression plasmid pLenti-GFP, the CHD7 overexpression plasmid pLenti-CHD7 or the CRISPR screen plasmid library was mixed with helper plasmids psPAX2 (Addgene, 12260) and pVSV-G (Addgene, 138479) together with Lipofectamine 2000 (Thermo Fisher Scientific, 11668019) according to the manufacturer’s instructions. The mixture was then added into the cell culture and incubated for 2 days. After the lentiviral transfection, the lentivirus supernatant was collected from the cell culture and filtered through a 0.45-µm strainer (Millipore, SLHAR33SS) to remove cell debris. The filtered lentivirus supernatant was concentrated using the Amicon Ultra-15 Centrifugal Filter Unit (Millipore, UFC910024), following the manufacturer’s instructions. The concentrated lentivirus supernatant was aliquoted and stored at −80 °C.

#### Generation of clonal A549-Cas9 cells

A549 cells (American Type Culture Collection (ATCC), CCL-185) were infected with lentivirus generated from the lentiCas9-Blast plasmid. Cells were under Blasticidin selection at 10 µg ml^−1^ until all untransduced cells were killed. The Blasticidin-selected, polyclonal cells were sorted into single cells by flow cytometry and plated onto 96-well plates. The single cells were clonally expanded and analyzed by immunofluorescence to verify Cas9 expression. The clonal selection was important to reduce the heterogeneity in the cell background in the screen.

#### Generation of BARC-FISH CRISPR screen cell library

Clonal A549-Cas9 cells were cultured to 60–80% confluency and transduced with the CRISPR screen lentivirus supernatant at a multiplicity of infection < 0.3 to ensure that most cells received only one genetic perturbation. Two days after the lentiviral infection, Puromycin (Thermo Fisher Scientific, A1113803) was added into the medium at 3 µg ml^−1^ to select the cells with resistance. Cells were under Puromycin selection for 10–12 days until the cell library was established. The cell library was then aliquoted into frozen stocks and stored in liquid nitrogen.

### Cell culture for cloning and imaging

A549 cells and the clonal A549-Cas9 cells (introduced in the section above, derived from the A549 cells) were maintained in F-12K medium (Corning, 10-025-CV) supplemented with 10% FBS (VWR, 97068-091) and 1% penicillin–streptomycin (Thermo Fisher Scientific, 15140122) at 37 °C in 5% CO_2_. hTERT RPE-1 cells (ATCC, CRL-4000) were maintained in DMEM:F-12 medium (ATCC, 30-2006) supplemented with 10% FBS (VWR, 97068-091) and 1% penicillin–streptomycin (Thermo Fisher Scientific, 15140122) at 37 °C in 5% CO_2_. To prepare imaging samples, coverslips (Bioptechs, 40-1313-03193) were disinfected by a 15-min ultraviolet treatment on each side before use. To culture cells for conducting the screen, A549-Cas9 cells transduced with the BARC-FISH screen library at the same passage were seeded onto the disinfected coverslips at 10% density in F-12K medium supplemented with 15% FBS and 1% penicillin–streptomycin and cultured for 6 days. The medium was also supplemented with 3 µg ml^−1^ Puromycin and 10 µg ml^−1^ Blasticidin (Thermo Fisher Scientific, R21001) to ensure sgRNA and Cas9 expression. Media were refreshed every 3–4 days. For lentivirus production, HEK-293FT cells were cultured in DMEM medium supplemented with GlutaMAX-I (Thermo Fisher Scientific, 10569-010), 1× MEM Non-Essential Amino Acids (Thermo Fisher Scientific, 11140-050), 10% FBS and 1% penicillin–streptomycin at 37 °C in 5% CO_2_, and were used within ten passages. All cells were routinely tested for mycoplasma contamination via Yale Molecular Diagnostics Laboratory.

### Detection of sgRNA–barcode associations in the cell library

#### NGS library preparation for mapping sgRNA–barcode associations

To determine the sgRNA–barcode correspondence in the screen library, the cell genomic DNA was PCR amplified for NGS. Because the total length of the sgRNA–barcode–UMI cassette is longer than the maximum sequencing length of NGS (300-bp paired-end sequencing on Illumina MiSeq), we generated two sequencing libraries: a sgRNA–barcode–UMI library and a barcode–UMI library. The sgRNA–UMI correspondence can be determined by the sgRNA–barcode–UMI library and the barcode–UMI correspondence can be determined by the barcode–UMI library. Then the sgRNA–barcode correspondence can be determined through their common UMI associated with both the sgRNA and the barcode on the same molecule. Briefly, the genomic DNA was extracted from the CRISPR screen cell library using the Zymo Quick-DNA Miniprep Kit (Zymo Research, D4068), following the manufacturer’s protocol. To achieve a sequencing coverage of >500, more than 2.5 million cells were harvested to cover the ~5,000 barcode varieties. We amplified all harvested genomic DNA and limited the number of amplification cycles to 20–23 to reduce amplification bias. For PCR amplification, each 50 µl reaction mixture was composed of 1 µg of genomic DNA, 500 nM of each forward and reverse primers, 5% dimethylsulfoxide and 1× NEBNext High-Fidelity PCR master mix (New England Biolabs, M0541L), following the manufacturer’s instructions. The PCR primers contained a flow-cell binding sequence, a sequencing index, a sequencing primer binding region and a 9–13-bp random sequence that improved the library diversity. After the reactions were completed, the PCR products were purified and concentrated using Zymo DNA Clean & Concentrator (Zymo Research, D4030), following the manufacturer’s instructions. Lastly, the concentrated products were run on a 2% agarose gel to validate the correct DNA amplicon size. The DNA library amplicons with the correct size were then gel purified and eluted with elution buffer (10 mM Tris-HCl, pH 8.5) or MilliQ water. The DNA libraries were sequenced on an Illumina MiSeq system in 2 × 300-bp format. The PCR primers were all purchased from IDT, and the sequences are provided in Supplementary Table 9.

#### sgRNA–barcode NGS analysis

As described above, we generated two sequencing libraries, a sgRNA–barcode–UMI library and a barcode–UMI library, and determined the sgRNA–barcode correspondence by the common UMI sequence. Briefly, the sequencing reads were filtered by read length and quality score. From the sgRNA–barcode–UMI library, protospacer and UMI sequences were extracted from the reads, and the protospacer–UMI lookup table was generated accordingly. The protospacer sequences were aligned with the sequences in the predesigned sgRNA oligonucleotide library such that the reads with improper protospacers were removed. From the barcode–UMI library, the UMI sequences were extracted and compared to the UMI found in the sgRNA–barcode–UMI library. Only reads with common UMIs were retained for further analysis. Because the read length from a single end did not cover the full length of the barcode, the sequencing read from each end only contained a partial barcode sequence. The digit values from the partial barcode sequences were extracted by BLAST^51^ against the predesigned digit sequences. The two decoded partial codes assigned to the two ends of the same sequence were then assembled into one full-length complete code by the overlapping region of the two partial codes. The partial codes that failed to overlap were excluded. The UMI–barcode lookup table was then established. The two lookup tables were merged to determine the sgRNA–barcode correspondence (codebook) through the shared UMIs. This codebook allowed analyses of the percentage of good codes (codes uniquely associated with one sgRNA) versus bad codes (codes associated with multiple sgRNAs; Extended Data Fig. 1d). The final codebook containing both the good codes that each uniquely associate with one sgRNA and the bad codes that each have multiple sgRNA projections is listed in Supplementary Table 10.

### Probe synthesis

The template oligonucleotide pool for synthesizing chromatin tracing primary probes was ordered from CustomArray (GenScript). We adopted a previously described probe synthesis workflow^52,53^ of limited-cycle PCR, in vitro transcription, reverse transcription, alkaline hydrolysis and column purification. The PCR primers and reverse transcription primers were ordered from IDT. The sequences of the template oligonucleotide library and primers are included in Supplementary Table 11.

### Imaging sample preparation

#### Geminin antibody staining

All steps were performed at room temperature unless otherwise described. Cells grown on coverslips were briefly rinsed with DPBS after media removal, and then fixed in 4% paraformaldehyde (PFA; Electron Microscopy Sciences, 15710) diluted in DPBS for 10 min followed by three DPBS washes. Cells were then permeabilized with 0.5% vol/vol Triton X-100 in DPBS for 10 min followed by three DPBS washes. After permeabilization, cells were blocked in blocking buffer (1% wt/vol bovine serum albumin (Sigma-Aldrich, A9647), 0.1% vol/vol Tween-20 in DPBS) supplemented with 0.1% vol/vol murine RNase inhibitor (MRI; New England Biolabs, M0314L) for 30 min, followed by primary antibody incubation for 1 h with a 1:100 dilution of anti-Geminin antibody (Abcam, ab195047) in blocking buffer supplemented with 1% vol/vol MRI. Unbound primary antibody was washed three times with 0.1% vol/vol Tween-20 in DPBS (DPBSTw), 5 min each, followed by a 1-h incubation with a 1:1,000 dilution of Alexa Fluor 488-labeled secondary antibody (Invitrogen, A11034) in blocking buffer supplemented with 1% vol/vol MRI. Starting from secondary antibody incubation, samples were protected from light during all steps. Excessive secondary antibody was washed off by washing three times, 5 min each with DPBSTw, and samples were post-fixed in 4% PFA in DPBS for 10 min followed by three DPBS washes. Post-fixed samples were next used for the BARC-FISH procedure in the screen experiments and for chromatin tracing primary probe hybridization in the validation experiments.

#### BARC-FISH

Helper probes and BARC-FISH linear and 5′-phosphorylated padlock probes were ordered from IDT. A total of 36 helper probes were pooled in an equimolar manner to a concentration of 5.6 µM for each probe. A total of 60 probes for BARC-FISH (30 linear probes and 30 padlock probes) were pooled in an equimolar manner to a concentration of 1.67 µM for each probe. Immediately before use, the pooled BARC-FISH probes were denatured at 90 °C for 2–5 min, and then cooled to room temperature. Samples with Geminin staining were pre-hybridized in 2× SSC buffer containing 20% vol/vol formamide and 0.1% vol/vol Tween-20 for 10 min, and then 50 μl of primary hybridization buffer was added to each sample, which contained 20% vol/vol formamide, 0.1 mg ml^−1^ salmon sperm DNA (Invitrogen, 15632-011), 800 nM each of the 60 BARC-FISH probes, 100 nM each of the 36 helper probes and 1% vol/vol MRI in 2× SSC. Samples were then incubated at 37 °C for 16–20 h. To remove excessive primary probes, samples were washed twice in 2× SSC containing 40% vol/vol formamide and 0.1% vol/vol Tween-20, 15 min each, followed by a third wash for 20 min at 37 °C in buffer containing 4× SSC, 1× DPBS, 0.1% vol/vol Tween-20 and 0.1% vol/vol MRI. Samples were then briefly rinsed twice with DPBSTw before the T4 ligation step. The T4 ligation mixture contained 0.2 mg ml^−1^ BSA (New England Biolabs, B9000S), 1 mM extra-supplemented dithiothreitol (Thermo Scientific, R0861), 1 mM extra-supplemented ATP (Thermo Scientific, R0441), 0.5 U μl^−1^ T4 DNA ligase (Thermo Scientific, EL0014) and 1% vol/vol MRI in 1× T4 ligase buffer (Thermo Scientific, EL0014). To perform the T4 ligation step, samples were incubated with 50 μl of T4 ligation mixture for 2 h, followed by two washes in DPBSTw. Samples were then incubated in a 30 °C water bath for 5 h with 50 μl of RCA mixture that contained 250 μM dNTP (New England Biolabs, N0447L), 1 mM extra-supplemented dithiothreitol, 0.2 mg ml^−1^ BSA, 1 U μl^−1^ phi29 enzyme (Thermo Scientific, EP0092) and 1% vol/vol MRI in 1× phi29 buffer (Thermo Scientific, EP0092). After the RCA step, samples were washed in DPBSTw, post-fixed with 4% PFA in DPBS for 30 min, and washed with DPBS. Post-BARC-FISH samples were used for the chromatin tracing primary probe hybridization in the screen experiments.

#### Chromatin tracing primary probe hybridization

Post-BARC-FISH samples (for screen experiments) or post-Geminin staining samples (for validation experiments) were briefly rinsed in DPBS, and then incubated with 0.1 M HCl diluted in water for 5 min. After two DPBS washes, samples were treated with 0.1 mg ml^−1^ RNase A (AB12023-00100) diluted in DPBS for 45 min at 37 °C, followed by two DPBS washes and one 2× SSC wash. Subsequently, samples were pre-hybridized in 2× SSC containing 50% vol/vol formamide and 0.1% vol/vol Tween-20 for 30 min and carefully dried by dipping on tissue paper to remove excessive pre-hybridization buffer. A total of 25 μl of hybridization buffer containing 50% vol/vol formamide, 20% vol/vol dextran sulfate (Millipore, S4030) and 4 μM (total concentration) chromatin tracing primary probes in 2× SSC was applied to a glass slide, and the sample coverslip was carefully flipped and placed on top so that the glass slide and coverslip ‘sandwiched’ the hybridization buffer, with the cells submerged into the hybridization buffer. The samples were then denatured on an 86 °C heat block (with a surface temperature of ~80 °C) for 3 min with the glass slide touching the heat block, and incubated in a humid chamber for 16–20 h at 37 °C. To remove excessive primary probes, samples were washed twice in a 60 °C water bath with 0.1% vol/vol Tween-20 in 2× SSC, for 15 min each, followed by a third 15-min wash with 0.1% vol/vol Tween-20 in 2× SSC at room temperature. Samples were then briefly rinsed with 2× SSC, and a 1:250,000 dilution of yellow-green fiducial beads (Invitrogen, F8803) in 2× SSC was applied to the samples and incubated for 10 min. Excessive beads were removed by two 2× SSC washes. Post-hybridization samples were used for automated sequential imaging.

### Automated sequential imaging

#### Imaging system setup

Four home-built fluorescence microscopes were used for image acquisition^52^. Each microscope had a Nikon Ti2-U body, a Nikon CFI Plan Apo Lambda ×60 oil objective lens (NA 1.40) and an automated focus-lock system^52,57^. One setup has identical lasers, light paths and filters as introduced previously^52^, and the image size was 1,536 × 1,536 pixels with a pixel size of 108 nm. For the other three setups, a Lumencor CELESTA light engine was used for illumination, with the following laser wavelengths: 405 nm, 477 nm, 546 nm, 638 nm and 749 nm. The 405-nm laser was used to excite and image the nuclear stain with DAPI. The 477-nm laser was used to excite and image the yellow-green fiducial beads and the Geminin stain. The 546-nm laser was used to excite and image ATTO 565 dye on secondary probes for BARC-FISH and chromatin tracing, and CF568 dye for total protein stain. The 638-nm laser was used to excite and image Alexa Fluor 647 dye on secondary probes for BARC-FISH and chromatin tracing. The 749-nm laser was used to excite and image Alexa Fluor 750 dye on secondary probes for BARC-FISH. A pentaband dichroic mirror supplied by Lumencor for the light engine was installed on the excitation path to direct the lasers to the sample, together with an ND0.6 neutral density filter to reduce the laser intensity. A pentaband emission filter supplied by Lumencor for the light engine and a Hamamatsu Orca Flash 4.0 V3 camera were each installed on the emission path. The image size was 2,048 × 2,048 pixels with a pixel size of 108 nm. A motorized *x*–*y* stage (SCAN IM 112 × 74, Marzhauser) was used to automatically image different fields of view (FOVs). Samples cultured on 40-mm no. 1.5 coverslips (Bioptechs, 40-1313-03193) were assembled in a Bioptech’s FCS2 flow chamber, mounted onto the microscope stage and connected with a previously described automated fluidic system^53,58^ for liquid handling during sequential hybridization and imaging.

#### Chromatin tracing sequential imaging

We conducted 17 and 14 rounds of three-color imaging for chromatin tracing of chr21 and chr22, respectively. During each round of hybridization, the sample was first incubated with 20% vol/vol ethylene carbonate (EC; Sigma-Aldrich, E26258) in 2×SSC containing two secondary probes (one labeled by ATTO 565 and one labeled by Alexa Fluor 647) for 25 min. The final concentration of each secondary probe was 6 nM, with the following exceptions for chr22 tracing to tune signal intensity differences: TAD 4 (0.5 nM), TAD 14 (12 nM), TAD 18 (12 nM) and TAD 26 (12 nM). In 6 initial screen replicates of the 17 total replicates, we used a TAD 4 probe concentration of 6 nM, which led to a bleedthrough of TAD 4 signal (labeled with Alexa Fluor 647 dye) into the TAD 18 fluorescent channel (546-nm laser channel). This bleedthrough issue was computationally addressed as described later (Supplementary Methods). The dye-labeled secondary probe sequences were selected from a previous study^20^. The secondary probes were ordered from IDT and their sequences are listed in Supplementary Table 12. Around 2 ml of wash buffer containing 20% vol/vol EC in 2× SSC was flowed through the chamber to remove unbound secondary probes, followed by 2 ml of anti-photobleaching oxygen-scavenging imaging buffer that contained 50 mM Tris-HCl pH 8.0, 5% wt/vol glucose, 2 mM Trolox (Sigma-Aldrich, 238813), 0.5 mg ml^−1^ glucose oxidase (Sigma-Aldrich, G2133) and 40 μg ml^−1^ catalase (Sigma-Aldrich, C30) in 2× SSC. During the overnight imaging process, the imaging buffer in the input tube was covered by a layer of mineral oil (Sigma-Aldrich, 330779-1L) to prevent oxidization. At each FOV, three *z*-stack images were taken sequentially with 638-nm, 546-nm and 477-nm laser illumination, with a step size of 200 nm, an exposure time of 0.4 s at each step and a total *z*-range of 7 μm. After all the FOVs were imaged for the current imaging round, the secondary probes were stripped off by slowly flowing 4 ml of 65% vol/vol formamide in 2× SSC for 10 min and incubating for an additional 5 min. Extra formamide was then removed by flowing 2 ml of wash buffer, and the next round of hybridization and imaging followed. To correct for color shift between 546-nm and 638-nm lasers, a no. 1.5 coverslip was coated with 100-nm Tetraspeck beads (Invitrogen, T7279) diluted at a 1:200 ratio in the imaging buffer, and *z*-stack calibration images were taken using the two lasers^20,52^.

#### BARC-FISH sequential imaging

For screen samples, we conducted ten rounds of four-color imaging to detect the barcode after chromatin tracing. During each round, the sample was first incubated for 25 min with 20% vol/vol EC in 2× SSC containing three secondary probes that correspond to the three values in each barcode digit, and the three probes were labeled with ATTO 565, Alexa Fluor 647 and Alexa Fluor 750, respectively. The concentrations of the Alexa Fluor 750-labeled and Alexa Fluor 647-labeled probes were 3 nM each, and the concentrations of ATTO 565-labeled probes were 6 nM each. Excessive probes were washed away by flowing 2 ml of wash buffer, followed by application of 2 ml of imaging buffer. At each FOV, four *z*-stack images were taken sequentially with 749-nm, 638-nm, 546-nm and 477-nm lasers. Each *z*-stack had a step size of 1.5 μm, an exposure time at each step of 0.4 s and a total range of 9 μm. After each imaging round, 11 ml of 90% vol/vol formamide in DPBS was flowed through the sample chamber for 28 min and then let stand for 100 s to remove the bound fluorescent probes. Excessive formamide was removed with 2 ml of wash buffer.

#### DAPI and total protein staining

For imaging screen samples, after BARC-FISH imaging was completed, the nuclei were stained by applying 3 ml of a 1:1,000 dilution of DAPI (Thermo Scientific, 62248) in 2× SSC in 4 min, and incubated for 3.5 min. The sample was then washed by applying 2 ml of wash buffer. Total protein stain was then conducted by diluting CF568-labeled succinimidyl ester (Biotium, 92131) at a ratio of 1:100,000 in water containing 0.1 M NaHCO_3_ and 25 mM Na_2_CO_3_, flowing 3 ml of the buffer through the sample chamber over 4 min, and incubating for 3.5 min. After further application of 2 ml wash buffer and 2 ml imaging buffer, three *z*-stack images were taken at each FOV sequentially with 546-nm, 477-nm and 405-nm lasers. Specifically, the *z*-stack of 546-nm and 477-nm lasers had the same step size, exposure time and total range as those used in BARC-FISH imaging, and the *z*-stack of 405-nm lasers had the same parameters as those used in chromatin tracing imaging. For imaging siRNA validation samples, DAPI staining was applied and imaged after chromatin tracing in a similar manner in the 477-nm and 405-nm channels, with parameters identical to those mentioned above.

### Reporting summary

Further information on research design is available in the Nature Portfolio Reporting Summary linked to this article.

## Online content

Any methods, additional references, Nature Portfolio reporting summaries, source data, extended data, supplementary information, acknowledgements, peer review information; details of author contributions and competing interests; and statements of data and code availability are available at 10.1038/s41592-025-02652-z.

## Supplementary information

Supplementary Information Supplementary Methods

Reporting Summary

Supplementary Table 1 Target gene summary. Three categories of target genes are included in the screen, as described in the table.

Supplementary Table 2 Top hits identified in the screen.

Supplementary Table 3 Digits and values for DNA barcode sequences in BARC-FISH screen, the corresponding linear and padlock probe sequences, and dye-labeled secondary probes.

Supplementary Table 4 Oligonucleotide sequences of helper probes.

Supplementary Table 5 Oligonucleotide sequences and PCR primers for barcode library assembly.

Supplementary Table 6 Protospacer sequences for gene targets in the screen, and the oligonucleotide sequences and PCR primers for sgRNA library assembly.

Supplementary Table 7 PCR primers for sgRNA–barcode CRISPR screen library cloning.

Supplementary Table 8 Primers for CHD7 overexpression plasmid cloning.

Supplementary Table 9 Oligonucleotide sequences for sgRNA–barcode sequencing library preparation.

Supplementary Table 10 BARC-FISH codebook determined by sequencing. The ‘Goodcodes’ worksheet contains good barcodes that each uniquely associate with an sgRNA, in which the first column represents the ten-digit barcode, and the second column represents the name of the sgRNAs listed in Supplementary Table 6. The ‘Badcodes’ worksheet contains barcodes that associate with multiple sgRNAs.

Supplementary Table 11 Template oligonucleotide sequences, PCR primers and reverse transcription primer used for chromatin tracing primary probe library synthesis.

Supplementary Table 12 Secondary probe sequences for chromatin tracing.

Supplementary Table 13 BARC-FISH screen results summary. Eight worksheets regarding eight phenotype analyses were included, and each worksheet consists of the following columns: sgRNA ID: sgRNA ranked alphabetically, representing their orders in Supplementary Table 6; sgRNA name: name of the sgRNAs as represented in Supplementary Table 6; copy number/cell number: number of traces (for chromatin-related phenotypes) or cells (for nuclear morphological properties) analyzed; *P*, FDR, log_2_fc and *z* indicate the *P* value, false discovery rate, log_2_ fold change and *z*-score of the analyzed phenotypes.

Supplementary Data S1 Backbone sequence used for barcode plasmid library construction.

Supplementary Data S2 Example barcode plasmid sequence.

Supplementary Data S3 Backbone sequence used for sgRNA plasmid library construction.

Supplementary Data S4 Example sgRNA plasmid sequence.

Supplementary Data S5 Backbone sequence used for CRISPR screen plasmid library construction.

Supplementary Data S6 Example CRISPR screen plasmid sequence.

Supplementary Data S7 Sequence of plasmid for CHD7 overexpression.

## Source data

Source Data Fig. 4 Full image of a western blot (presented in Fig. 4a).

Source Data Extended Data Fig./Table 5 Full image of a western blot (presented in Extended Data Fig. 5a).

Source Data Extended Data Fig./Table 6 Full image of western blots (presented in Extended Data Fig. 6a,b).

## Data Availability

Raw sequencing data of the CRISPR screen cell library and analyzed imaging data generated from this study are available for download at https://campuspress.yale.edu/wanglab/BARCFISH/. Raw sequencing data have been deposited into the NCBI Sequence Read Archive database under accession no. PRJNA1225422. Raw imaging data are available from the corresponding author upon request and are not deposited online due to the prohibitively large size. The Human Protein Atlas dataset can be accessed at https://www.proteinatlas.org/. Source data are provided with this paper.

## Code availability

All original code generated for this study are available for download at https://campuspress.yale.edu/wanglab/BARCFISH/ and on Zenodo via 10.5281/zenodo.14227128 (ref. ^59^). Open-source codes for imaging data collection are available at https://github.com/ZhuangLab/storm-control/.
