# Supplementary material for: Perturb-tracing enables high-content screening of multi-scale 3D genome regulators
Source: Nat Methods. 2025 Apr 10;22(5):950–61. doi: 10.1038/s41592-025-02652-z (PMC12074983; doi:10.1038/s41592-025-02652-z)
Supplement: Supplementary file 1 — Supplementary Methods [file 41592_2025_2652_MOESM1_ESM.pdf]

nature methods

Supplementary information https://doi.org/10.1038/s41592-025-02652-z

Perturb-tracing enables high-content screening of multi-scale 3D genome regulators

In the format provided by the authors and unedited

Supplementary Methods

Plasmid construction for CHD7 and GFP overexpression

The GFP lentiviral overexpression vector, pLenti-GFP, was purchased from OriGene (OriGene, PS100093). To construct the CHD7 lentiviral overexpression construct, we cloned the human CHD7 open reading frame (ORF) into the same vector upstream of GFP, together with a P2A sequence placed between CHD7 and GFP for self-cleavage. Specifically, the CHD7 ORF was PCR amplified from a plasmid (Addgene, 89460) using Phusion High-Fidelity Master Mix (NEB, M0531L) following the manufacturer's instructions. The P2A sequence was added to the 3' end via reverse primer. Then, DpnI (NEB, R0176S) was directly added to the PCR mixture and incubated at 37℃ for 1 hour to digest the plasmid template. The PCR products of correct size were confirmed by agarose gel electrophoresis and gel purified. To prepare the backbone, pLenti-GFP was digested by restriction enzymes AsiSI (NEB, R0630S) and MluI-HF (NEB, R3198S), and dephosphorylated by Shrimp Alkaline Phosphatase (NEB, M0371S) at 37℃ for 3.5 hours. The resulting backbone was gel purified, mixed with the purified PCR products, and subjected to Gibson Assembly using NEBuilder HiFi DNA Assembly Master Mix (NEB, E2621L) following the manufacturer's instructions. The reaction mixture was then column purified and transformed into chemically competent E. coli Stbl3 (ThermoFisher, C737303) following the manufacturer's instructions. The transformed cells were plated onto LB agar plates containing 34 µg/mL chloramphenicol and incubated overnight at 37℃. Several clones were picked and individually inoculated into LB liquid medium overnight at 37℃, ~225 rpm. The plasmids of individual clones were extracted using QIAprep Spin Miniprep Kit (Qiagen, 27106) following the manufacturer's instructions, and the correct sequence was confirmed by Sanger sequencing. The primer sequences for cloning and Sanger sequencing are provided in Supplementary Table 8. The plasmid sequence with annotations of the reconstructed CHD7 lentiviral overexpression vector, pLenti-CHD7, is included in Supplementary File S7.

Cell line construction and culturing

Generation of CHD7 and GFP OE cell line

Clonal A549-Cas9 cells were infected with the concentrated lentivirus produced from the overexpression plasmid pLenti-GFP or pLenti-CHD7. Puromycin selection started two days after lentiviral infection at a concentration of 3 µg/mL in growth media, and lasted for 5-6 days until the stable cell lines were established.

Human ESC culture

H1 human ESC lines (WiCell WA01, from Yale Stem Cell Core Facility) were cultured in mTeSR1 (Stem Cell Technologies) in feeder free conditions on 1x hESC certified matrigel (Corning 354277) with daily media changes for the duration of culture. Cells were passaged weekly with dispase maintaining ESCs in colonies (Stem Cell Technologies 07913). Cells were weeded manually whenever differentiation was observed. When ready to differentiate cells, ESCs were detached and made into a single cell suspension using Accutase (Stem cell technologies 07920).

Generation of CHD7 knockdown H1 hESC line

With the Yale Functional Genomics Core, lentiviruses were produced by co-transfecting HEK-293FT cells with packaging vectors psPAX2 (Addgene plasmid #12260) and pMD2.G (Addgene

plasmid #12259) together with lentiviral transfer constructs. Viral supernatant was collected 48h and 72h after transfection and filtered with a 0.45-µm filter. Viral supernatant, either Sigma Mission SHC002 nontargeting shRNA, to be referred to as shControl, or shCHD7 (Sigma Mission shRNA TRCN0000016408) was diluted 1:5 into mTeSR1 and plated onto cells for 24 hours. After infection, the viral media was replaced with mTeSR1 for an additional 24 hours before beginning selection. Cells were selected in 10 µg/mL puromycin with daily media changes for 7 days, at which point the cells were taken off selection. Prior kill curves in house have determined the kill time of 10 µg/mL puromycin in wild type cells to be 2 days. A well of uninfected cells was included on each plate as a control to verify 100% cell death without puromycin resistance. Cells were passaged or frozen after expansion following selection. Cells were frozen in 40% Knockout serum replacement (Thermofisher 10828028) 10% DMSO and 50% mTeSR1 and were slow frozen overnight in an isopropanol bath.

Generation of Neural Crest
Knockdown and control transfected hESCs were differentiated to neural crest using the STEMdiff Neural Crest Differentiation Kit (Stem cell technologies 08610) exactly according to manufacturer's specifications. In brief, hESCs were dissociated into single cells with Accustase and seeded as a single cell suspension in complete neural crest media supplemented with 10 µM Y-27632 (Dihydrochloride) (Stem cell technologies 72304). Neural crest media was changed daily for 6 days. Afterwards, cells were passaged with accutase onto coverslips coated with Matrigel. These coverslips were then fixed and treated as described for chromatin tracing and IF 1-2 days after passaging onto coverslips and were not maintained long term but were generated fresh as needed from frozen hESC stocks.

<u>Validation of CRISPR knockout efficiency</u>
To validate the CRISPR knockout efficiency, we constructed individual CRISPR knockout cell lines for selected sgRNAs and utilized NGS of genomic DNA to analyze the frameshift indel mutations. The cloning procedure of individual sgRNA plasmids was similar to previously described in "sgRNA plasmid library construction", except that we directly ordered double-stranded sgRNA fragments from IDT for Gibson Assembly. Cell line generation procedure was identical to "Generation of BARC-FISH CRISPR screen cell library". We then performed genomic DNA extraction and PCR amplification following a procedure similar to previously described in "Next-generation sequencing (NGS) library preparation for mapping sgRNA-barcode associations". The amplified region was 250-450 bp centered around the sgRNA targeting site. The PCR amplicons were sequenced on Illumina MiSeq system in 2×250 bp format, achieving ~50,000 reads per sgRNA. The indel rate analysis was performed and delivered by Genewiz.

<u>siRNA knockdown</u>
siRNA in A549 cells. The clonal A549-Cas9 cells used to construct the screen cell library were seeded at 6% density one day before siRNA transfection. The siCHD7 single knockdown experiments were conducted using Dharmacon ON-TARGETplus siRNA pools and DharmaFECT transfection reagent (Dharmacon, T-2001-03) following the manufacturer's protocol. Briefly, for each 10-cm dish, 3 µL of 100 µM siCtrl (Dharmacon, D-001810-10) or siCHD7 (Dharmacon, L-025947-01) was diluted to 1.2 mL Opti-MEM reduced-serum medium (Thermo Fisher Scientific, 31985-070). In a separate tube, 24 µL of transfection reagent was diluted to 1.2 mL Opti-MEM reduced-serum medium. The diluted siRNA and the diluted

transfection reagent were incubated for 5 minutes at room temperature. Then the diluted siRNA was added into the diluted transfection reagent and the mixture was incubated for 20 minutes at room temperature. Lastly, the antibiotics-free growth medium was added to a final volume of 12 mL. Cells were cultured with the 12 mL transfection medium at 37°C for 96 hours and used for follow-up analysis, including Western blot and chromatin tracing.

siRNA in hTERT-RPE1 cells. The hTERT-RPE1 cells were seeded at 6% density one day before siRNA transfection. The same siRNA pools for siCtrl and siCHD7 in A549 RNAi experiments and RNAiMAX transfection reagents (Invitrogen, 13778-075) were used. Transfection was done following RNAiMAX manufecturer's protocol with 50nM final concentration of siCtrl or siCHD7 siRNA. After siRNA transfection, cells were cultured with the transfection mix for 4 days before downstream assays.

Western blot

Cells were trypsinized by TrypLE Express (Thermo Fisher Scientific, 12605-010) and pelleted through centrifugation at 500g for 5 minutes at 4°C. To enrich the nuclear proteins, cell nuclei were isolated and lysed using NE-PER Nuclear and Cytoplasmic Extraction Kit (Thermo Fisher Scientific, 78833) following the manufacturer's instructions. For hESCs and human neural crest progenitors, protein was extracted using RIPA buffer (Thermo Scientific 89900). The reagents were supplemented with 1× protease inhibitors (Thermo Fisher Scientific, 87786) to prevent the protein degradation during extraction. The protein concentrations were quantified using Pierce BCA protein assay kit (ThermoFisher, 23225) following the manufacturer's instructions. The protein was denatured at 95-100°C for 5 minutes in 1× NuPAGE LDS sample buffer (ThermoFisher, NP0007) containing 10% v/v β-Mercaptoethanol (Millipore Sigma, M6250). For detection of CHD7 protein, the denatured nuclear extract was subjected to electrophoresis on a precast 3-8% Tris-Acetate polyacrylamide gel (Thermo Fisher Scientific, EA0378BOX) in 1× Tris-Acetate SDS running buffer (Thermo Fisher Scientific, LA0041) at 100V for 2.5 hours. For detection of PCBP1, ZNF114 and Actin, the protein electrophoresis was performed using 4-15% precast polyacrylamide gels (Biorad, 4568084) in 1× Tris/Glycine/SDS buffer (diluted from Biorad, 1610732) at 100V for 1.5 hours. For hESCs and human neural crest progenitors, the protein electrophoresis was performed using 4-20% precast polyacrylamide gels (Biorad, 4568095) in 1× Tris/Glycine/SDS buffer (diluted from Biorad, 1610732) at 100V for 2.5 hours for all proteins. The proteins were transferred from the gel onto a nitrocellulose membrane (ThermoFisher, IB301032) using either iBlot2 dry blotting system (Thermo Fisher Scientific, IB21001S) or wet transfer using the Biorad Mini Trans-Blot Cell following the manufacturer's instructions. Wet transfer buffer was 20% methanol, 200 mM glycine, and 250 mM Tris. The membrane was blocked in 5% BSA in TBST for 1 hour at room temperature with gentle shaking. Primary antibodies against human CHD7 (Thermo Fisher Scientific, PA5-72964), HSP90 (CST 4874S), Sox10 (CST 89356S), PCBP1 (sc-137249), ZNF114 (NBP1-81181) and Actin (Abcam, ab179467) were incubated with the membrane at recommended concentrations at 4℃ overnight with gentle shaking. The following day, the membrane was washed three times in TBST for 5 minutes each, followed by incubation with horseradish peroxidase-conjugated secondary antibodies (Abcam, ab6721 or ab6789) diluted at 1:3000 ratio in 5% non-fat milk in TBST for 1 hour at room temperature. The blots were then washed three times in TBST for 5 minutes each. PCBP1, ZNF114 and Actin blots were treated with SuperSignal West Pico PLUS Chemiluminescent Substrate (Thermo Fisher Scientific, 34580) and CHD7 blot was treated with

SuperSignal West Femto Maximum Sensitivity Substrate (ThermoFisher, 34094) for 2-5 minutes at room temperature. The blots were imaged using a CCD camera-based imager, with AlphaView software (4.1.4) in the ProteinSimple FluorChem E system or the Image Lab Touch Software (3.0.1.14) in the Bio-Rad ChemiDoc MP Imaging System. To detect Actin alongside PCBP1 or ZNF114 on the same membrane, the membrane was first incubated with antibodies against PCBP1 or ZNF114 and imaged. It was then stripped using stripping buffer (Thermo Fisher Scientific, 46430) for 15 minutes at room temperature before incubation with the Actin antibody for detection as mentioned above.

Immunofluorescence of Cas9

Cells were fixed in 4% PFA in DPBS for 10 minutes and washed twice with DPBS. Cells were then permeabilized in 0.5% v/v Triton X-100 in DPBS for 10 minutes and washed twice with DPBS. Cells were blocked in blocking buffer (1% w/v BSA and 0.1% v/v Tween-20 in DPBS) for 30 minutes at room temperature with gentle agitation. Then the cells were incubated for 1 hour at room temperature with 1:500 diluted anti-Cas9 primary antibody (Sigma, SAB4200701-25UL, for detection of Cas9 expression) in blocking buffer, followed by three DPBS washes for 5 minutes each. The cells were incubated with 1:1000 diluted Alexa Fluor 647-labeled secondary antibody (Thermo Fisher Scientific, A21237) in blocking buffer for 1 hour at room temperature, followed by three DPBS washes for 5 minutes each. Starting from incubation with secondary antibody, the samples were covered from the light with aluminum foil.

chr22 whole chromosome paint

To generate probes for chr22 whole chromosome paint, we synthesized chr22 tracing library with Alexa Fluor 647 labeled reverse transcription primer, so that all the TADs of chr22 can be directly stained together. Geminin staining was first performed after cell fixation as described in *Geminin antibody staining*. Next, chr22 was stained with the dye-labeled whole chromosome paint probe library following the same procedure as described in *Chromatin tracing primary probe hybridization*.

Data analysis

Determination of chromatin traces in 3D. Image and phenotype analyses were performed using MATLAB version R2020a. We adopted a previously reported pipeline to determine the 3D chromatin traces[52]. Briefly, to correct the drift between sequential chromatin tracing secondary hybridization rounds, we determined the 3D positions of fiducial beads during each round of hybridization by fitting their z-stack images to Gaussian functions in 3D. We then fitted the center position of each DNA FISH foci in 3D using the same algorithm, and the sample drift (represented by bead movements) was subtracted from the 3D position of each DNA locus. The drift-corrected DNA loci positions were then linked into traces based on their expected spatial proximity in each chromosome territory. To remove false traces generated from non-specific target objects or genomic DNA released from dead cells, linked traces were excluded if 1) they located outside of the nucleus, represented by 2D-projected DAPI staining patterns, or 2) their radii of gyration was smaller than 0.5 μm. In a small number of datasets (see *Chromatin tracing sequential imaging* section for explanation), we noticed that due to extra-strong labeling of TAD4, signal from TAD4 (638-nm channel) could be excited with 546-nm laser and thus be misidentified as TAD18. To correct this in the affected datasets, after the traces were linked, we calculated the 3D distance between TAD4 and TAD18 on each trace in those datasets. If the

distance was smaller than 100 nm, the 3D position recognized as TAD18 were regarded as bleedthrough from TAD4, and the 3D positions of TAD18 were assigned to NaN.

BARC-FISH pattern extraction. The decoding process consisted of the following steps: 1) drift correction, 2) identifying all possible BARC-FISH patterns for each value of each barcode digit, 3) filtering mis-identified BARC-FISH patterns based on intensity thresholding, and 4) assigning the digit identity to each cell. To correct the drift between sequential BARC-FISH imaging rounds, the center positions of the fiducial beads were determined by fitting to Gaussian functions in 2D, and the movement was subtracted from BARC-FISH images. To extract the BARC-FISH patterns for each value in each digit, BARC-FISH images were first projected along z dimension with average intensity projection, and filtered by a 2D median filter and a Gaussian low-pass filter. The image background was derived by image opening with a disk-shaped structural element with a 20-pixel radius, and the background was subtracted. Potential BARC-FISH areas were identified by binarizing the background-subtracted image and selecting objects that were larger than 50 pixels. Due to the strong labeling intensity of BARC-FISH foci, bleedthrough was often seen in channels excited with lasers of shorter wavelength. To correct this, the bleedthrough of Alexa Fluor 647 into 546-nm channel was identified by the shared areas between 546-nm and 638-nm channel in the same BARC-FISH round, and the areas were subtracted from the 546-nm BARC-FISH pattern. Similarly, the bleedthrough of Alexa Fluor 750 into 638-nm channel was subtracted from the 638-nm BARC-FISH pattern. Regions that were positive for all three channels (546-nm, 638-nm and 749-nm) during one BARC-FISH round were subtracted from all three channels because such regions could be due to broad-spectrum autofluorescent objects or non-specific binding of all probes to the same sticky objects. These steps generated high-fidelity binary masks for BARC-FISH signals. To further increase the reliability of our identified BARC-FISH foci masks, we applied the binary masks to the median- and Gaussian-filtered BARC-FISH images collected in 15-20% of the FOVs collected in each screen dataset. For each value of each digit, we calculated the normalized intensity ([total intensity] / [pattern size]) of each BARC-FISH focus identified through the binary mask, and sorted the normalized intensity. A normalized intensity cutoff was then derived for each value of each digit, and applied to all the FOVs in the dataset to only include the BARC-FISH patterns of which the normalized intensity was above this threshold.

Cell segmentation and decoding. Drift between the DAPI/total protein stain imaging round and previous rounds were corrected using the same algorithm as in BARC-FISH pattern extraction. Z-stacks of DAPI and total protein stain was projected along the z dimension with max and average intensity projections, respectively. A median filter was applied to the z-projected DAPI images. To remove the bleedthrough of the fiducial beads pattern into total protein stain, total protein images was eroded by a disk-shaped structural element with a radius of 5 pixels. We then performed watershed-based cell segmentation of the total protein stain profile using DAPI patterns as foreground markers. To determine the barcode identity of each cell, for each digit, we calculated the total pixel counts from BARC-FISH pattern of each of the three values, value 0 (546-nm channel), 1 (638-nm channel) or 2 (749-nm channel), and assigned the value with the largest pixel count to the digit of the cell. Cells with BARC-FISH signal in none of the 3 values were assigned a fourth value of 10000. We then calculated the Hamming distance between the current decoded barcode to those in the codebook. A barcode was decoded/corrected if: 1) the current decoded barcode has a unique closest match in the codebook, 2) this unique closest

match is a good code (a code that uniquely associates with only one sgRNA), and 3) the Hamming distance between the decoded barcode and the closest match was not larger than 1 (maximum 1 mismatching digit allowed). The cell carrying the decoded/corrected barcode was assigned the corresponding sgRNA identity.

Cell cycle stage classification. We only included G1-phase cells in phenotypic analyses of both the screen and the validation. G1 cells were indicated by an absence or low intensity of Geminin stain in the nucleus. The foreground DAPI marker generated for the watershed-based cell segmentation was used as a binary mask of nuclear regions. Two methods were used to determine the cell cycle status: 1) We calculated the normalized Geminin intensity for each cell by dividing the total nuclear Geminin fluorescence intensity by the area of the nucleus, and empirically determined the threshold for each imaging experiment between Geminin-positive and Geminin-negative cells based on the distribution of the normalized intensity. Cells of which the normalized nuclear Geminin intensity was below the threshold were included in phenotypic analyses. This method was used for 13 out of 17 screen experiments and all validation experiments. For 4 early replicates, due to area-to-area variation of Geminin staining, a uniform normalized nuclear Geminin intensity could not be applied to all FOVs for G1 cell identification. For these 4 replicates, we determined the cutoff for G1 versus non-G1 cells by calculating the ratio between the normalized Geminin intensity in the nucleus and the whole cell area (determined by watershed boundaries) for each cell, and empirically determined the threshold for each dataset based on the distribution of this ratio.

Analysis of adjacent TAD distance and contact. Supplementary Table 13 includes the exact sample size (*n*), the p-value, false discovery rate (FDR) and log2 fold change (log2fc) value for each analyzed sgRNA and for each phenotype analyzed in the screen. We grouped traces from the same sgRNA (determined by the cell barcode detected via BARC-FISH) across different datasets. To increase the statistical power in identifying novel hits, traces from all 412 observed sgRNAs in the screen were pooled as control (termed as "whole population"), and 341 sgRNAs with at least 40 chromatin traces were compared to the whole population. For our non-targeting gRNA controls, we found 5 out of the 8 observed non-targeting gRNAs showed no significant phenotype in any category. We combined the five into a single control group and reported it in Supplementary Table 13 as a "combined non-targeting control". The combined non-targeting control gRNA group also showed no significant phenotype. Adjacent TADs were defined as TADs that were next to each other along the genomic map. To compare adjacent TAD distance, we calculated the distance between each pair of adjacent TADs on each trace, and generated a mean adjacent TAD distance profile for each sgRNA by calculating the mean distance for each pair of adjacent TADs. We then conducted two-sided Wilcoxon signed rank test in MATLAB to compare the mean adjacent TAD distance between each sgRNA and the control group, calculated FDR from the resulting p values, and selected top hits that 1) have FDRs<0.1, 2) have the largest absolute values of log2fc, and 3) have protein localization in the cell nucleus based on The Human Protein Atlas (proteinatlas.org) to prioritize potential direct regulators of 3D genome. To calculate the log2 fold change in Fig. 2a, we divided the mean adjacent TAD distance between the same pair of TADs from the sgRNA by that from the control group, calculated the log2 fold change value for each TAD pair, and averaged the log2 fold change values to generate the log2 fold change for each sgRNA. Log2 fold change was calculated in a similar manner in Fig. 2j by averaging the log2 fold change value of all pairwise comparisons.

Determination of compartment identity of TADs. We assigned A/B compartment identity to the TADs using a previously introduced algorithm[52]. Briefly, we fitted a power-law function to the data points of the mean inter-TAD spatial distances versus their genomic distances, which yielded the expected inter-TAD spatial distance for each pair of TADs according to their genomic distance. We then normalized the observed mean inter-TAD spatial distance by the expected spatial distance, yielding a normalized inter-TAD distance matrix. We then calculated the Pearson correlation coefficient between each pair of rows/columns in this matrix, generating a Pearson correlation matrix. We next applied principal component analysis to the Pearson correlation matrix, and took the coefficients of the first principal component as compartment scores. The compartment score profiles were cross-validated with published ChIP-seq profiles[59,60], so that the compartment A regions are enriched in active histone modifications, while compartment B regions are enriched in repressive histone modifications. If the trend was opposite, the signs of the compartment scores were flipped. In the CRISPR screen, traces from the whole population were pooled to generate the compartment identity, which was applied to different perturbations when evaluating long-range AA, AB and BB compartment contacts (Fig. 2d-i).

Analysis of long-range contact in different compartments. Long-range contacts were defined as the spatial contact events between TAD pairs that are not adjacent TADs on the genomic map. We used the same distance threshold for calling contacts as that used in the adjacent TAD contact, and calculated the numbers of AA, AB and BB long-range TAD contact events per trace. In Fig. 2d-i, m, Fig. 3 and Fig. 4m, AB compartment profile generated from all traces were used to define A and B compartments. In Fig. 4e, Extended Data Fig. 3d, and 4e, AB compartment profile of the according control group were used to define A and B compartments.

Analysis of overall chromosome compaction. We calculated the inter-TAD distances between all pairs of TADs on chr22 for each sgRNA, and generated mean inter-TAD distance matrix for each sgRNA by calculating the mean distance between each pair of TADs. We then performed two-sided Wilcoxon signed rank test between the mean inter-TAD distances of each sgRNA and control, and calculated FDR and log2 fold change values.

Analysis of nuclear morphological properties. To increase the statistical power of the screen, only sgRNAs with more than 10 nuclei were included in the screen analyses (Fig. 5a,e). We compared the 3D coefficient of variation of nuclear stain intensity (nuclear intensity unevenness) and nuclear sphericity between each sgRNA and control. To abstract the nucleus shape in 3D, we first calculated a normalization factor of each FOV by selecting the max intensity of the median-filtered z-stack images of the current FOV. We normalized the max z-projected DAPI image by this normalization factor, and calculated a background by applying adaptive thresholding on the normalized image using a sensitivity of 0.4. We then processed each layer of the z-stack, normalized the median-filtered current slice of z-stack image by the normalization factor, and binarized it using the threshold generated from the previous adaptive thresholding step. Only binary objects at each slice that were larger than 5,000 pixels were retained. Partial nuclei at the edges of each FOV were excluded. We then matched the cell identity to each segmented 3D nucleus, and analyzed the coefficient of variation of voxel intensity within each nucleus of each sgRNA. The nuclear volume and surface area were calculated by the *regionprops3* function in MATLAB. The nuclear sphericity was determined by the following equation:

$$Sphericity = \frac{\pi^{\frac{1}{3}}\,(6 * Volume)^{\frac{2}{3}}}{Surface\ area}$$

A sphericity of 1 indicates the shape is a perfect sphere. A sphericity smaller than 1 indicates reduced roundness. Sphericity values exceeding 1 due to inaccuracy of surface area calculation by the *regionprops3* function were excluded from the analysis. For volcano plots, p values were calculated by unpaired t test followed by FDR calculation (Fig. 5a,e). For box plots, p values were calculated by two-sided Wilcoxon rank sum test (Fig. 5b,f).

Calculation of radius of gyration. We only retained traces with more than 80% of TADs detected for analyzing this phenotype. The radius of gyration was defined as the root mean square of the distance between each TAD to the centroid of the trace, as shown in the following equation:

$$Radius\ of\ gyration = \sqrt{\frac{1}{n}\sum_{i=1}^{n} d_i^2}$$

where n is the number of detected TADs in the given trace, and d_i represents the spatial distance between TAD i and the centroid of the trace.

Quantification of polarized organization of A-B compartments. Only traces with more than 80% of TADs detected were included in this analysis. We used a previously developed algorithm[52] to quantify the polarized organization of A-B compartments, yielding a polarization index. Briefly, we constructed 3D convex hulls for all A compartment TADs and B compartment TADs on each individual trace, with the *convhull* MATLAB function. We then calculated the volume of these two hulls (referred to as V_A and V_B, respectively) and the volume of the overlapped spaces between A and B hulls (referred to as V_S). Polarization index was defined as:

$$Polarization\ index = \sqrt{\left(1 - \frac{V_S}{V_A}\right)\left(1 - \frac{V_S}{V_B}\right)}$$

A polarization index of 1 indicates that the A and B compartments are completely spatially separated from each other in a side-by-side manner. A polarization index of 0 indicates that A and B compartments completely overlap, or one is surrounded by the other. The A-B compartment profile of the according treatment was used to calculate polarization indices for each condition.

Quantification of chr22 whole chromosome paint. To segment chr22 territories, chromosome paint images were first projected along z dimension with max intensity projection and filtered by a 2D median filter. Next, two rounds of background removal were performed. First, background images were generated using the adaptthresh function in MATLAB with neighborhood size of 201 pixels. The background was removed by division with the background image and the maximum pixel value of the resultant image was normalized to 1. Second, background was derived by image opening with a disk-shaped structuring element with a 5-pixel radius, and the background was subtracted. Then, images were further processed with close-by-reconstruction and image closing with a disk-shaped structuring element with a radius of 10 and 3 pixels respectively. Finally, chr22 territories were identified by binarizing the image with an intensity

value threshold of 0.03. DAPI segmentation was also performed and G1 phase cells were selected based on Geminin signal. chr22 territory area per cell was quantified for comparison.

Chromatin polymer simulation

A Monte Carlo simulation of chromatin conformation with a lattice polymer model was adapted from a previous report[52] with the following modifications: 1) The polymer contained 30 monomers; 2) the maximum allowed distance between adjacent monomers along the polymer was 3; 3) to calculate the energy of a polymer conformation, we included only one type of interaction energy – any pair of monomers with a distance closer than 2 would incur an energy loss of K. To build a bounding envelope of the simulated chromatin conformation, at each monomer, we created a sphere with a radius of 15 pixels, the centroid of which is the current monomer. We then filled and smoothened the “dilated” 3D shape by function *imfill* and *imclose* in MATLAB. We generated the isosurface of the dilated shape by MATLAB function *isosurface* to define the bounding envelope of the chromatin conformation for sphericity calculation and visualization in Fig. 5l.
