# Supplementary material for: Perturb-tracing enables high-content screening of multi-scale 3D genome regulators
Source: Nat Methods. 2025 Apr 10;22(5):950–61. doi: 10.1038/s41592-025-02652-z (PMC12074983; doi:10.1038/s41592-025-02652-z)
Supplement: Supplementary file 2 — Reporting Summary [file 41592_2025_2652_MOESM2_ESM.pdf]

nature portfolio

Corresponding author(s): Siyuan Wang

Last updated by author(s): Feb 20, 2025

Reporting Summary

Nature Portfolio wishes to improve the reproducibility of the work that we publish. This form provides structure for consistency and transparency in reporting. For further information on Nature Portfolio policies, see our Editorial Policies and the Editorial Policy Checklist.

Statistics

For all statistical analyses, confirm that the following items are present in the figure legend, table legend, main text, or Methods section.

n/a	Confirmed	
☐	☒	The exact sample size (*n*) for each experimental group/condition, given as a discrete number and unit of measurement
☐	☒	A statement on whether measurements were taken from distinct samples or whether the same sample was measured repeatedly
☐	☒	The statistical test(s) used AND whether they are one- or two-sided<br>*Only common tests should be described solely by name; describe more complex techniques in the Methods section.*
☐	☒	A description of all covariates tested
☐	☒	A description of any assumptions or corrections, such as tests of normality and adjustment for multiple comparisons
☐	☒	A full description of the statistical parameters including central tendency (e.g. means) or other basic estimates (e.g. regression coefficient) AND variation (e.g. standard deviation) or associated estimates of uncertainty (e.g. confidence intervals)
☐	☒	For null hypothesis testing, the test statistic (e.g. *F*, *t*, *r*) with confidence intervals, effect sizes, degrees of freedom and *P* value noted<br>*Give P values as exact values whenever suitable.*
☒	☐	For Bayesian analysis, information on the choice of priors and Markov chain Monte Carlo settings
☒	☐	For hierarchical and complex designs, identification of the appropriate level for tests and full reporting of outcomes
☐	☒	Estimates of effect sizes (e.g. Cohen's *d*, Pearson's *r*), indicating how they were calculated

Our web collection on statistics for biologists contains articles on many of the points above.

Software and code

Policy information about availability of computer code

Data collection	Open-source codes for imaging data collection are available at https://github.com/ZhuangLab/storm-control. To capture the Western blot images, AlphaView software (4.1.4) in the ProteinSimple FluorChem E system or the Image Lab Touch Software (3.0.1.14) in the Bio-Rad ChemiDoc MP Imaging System was used.
Data analysis	All original codes generated for this study are available for download at https://campuspress.yale.edu/wanglab/BARCFISH and https://doi.org/10.5281/zenodo.14227128. ProbeDealer was used for probe design. MATLAB (R2020a) was used for analysis of all imaging data and next-generation sequencing data.

For manuscripts utilizing custom algorithms or software that are central to the research but not yet described in published literature, software must be made available to editors and reviewers. We strongly encourage code deposition in a community repository (e.g. GitHub). See the Nature Portfolio guidelines for submitting code & software for further information.

Data

Policy information about availability of data

All manuscripts must include a data availability statement. This statement should provide the following information, where applicable:

- Accession codes, unique identifiers, or web links for publicly available datasets
- A description of any restrictions on data availability
- For clinical datasets or third party data, please ensure that the statement adheres to our policy

Raw DNA sequencing data of the CRISPR screen cell library and analyzed imaging data generated from this study are available for download at https://campuspress.yale.edu/wanglab/BARCFISH. Raw sequencing data have been deposited into the NCBI Sequence Read Archive database under accession no. PRJNA1225422. Raw imaging data are available from the corresponding author upon request and are not deposited online due to prohibitively large size. The Human Protein Atlas dataset can be accessed at https://www.proteinatlas.org.

Research involving human participants, their data, or biological material

Policy information about studies with human participants or human data. See also policy information about sex, gender (identity/presentation), and sexual orientation and race, ethnicity and racism.

Reporting on sex and gender	This information is not involved in this study.
Reporting on race, ethnicity, or other socially relevant groupings	This information is not involved in this study.
Population characteristics	This information is not involved in this study.
Recruitment	This information is not involved in this study.
Ethics oversight	This information is not involved in this study.

Note that full information on the approval of the study protocol must also be provided in the manuscript.

Field-specific reporting

Please select the one below that is the best fit for your research. If you are not sure, read the appropriate sections before making your selection.

☒ Life sciences ☐ Behavioural & social sciences ☐ Ecological, evolutionary & environmental sciences

For a reference copy of the document with all sections, see nature.com/documents/nr-reporting-summary-flat.pdf

Life sciences study design

All studies must disclose on these points even when the disclosure is negative.

Sample size	Sample sizes are reported in the manuscript and figure legends. The number of datasets we collected was determined to be the largest number we could screen currently with available personnel and equipment in the study period.
Data exclusions	In imaging analyses, non-G1 phase cells were excluded to avoid cell cycle as a confounding factor.
Replication	The image-based screen contains 17 biological replicates, and all attempts of the 17 replicates were successful.
Randomization	To analyze and evaluate the A-B compartment polarization index, randomized controls were generated using a previously published method. Please see the detailed description in the Methods of the manuscript. Same traces were used for experimental and randomized controls, with no grouping involved and no covariate identified.
Blinding	Not applicable. Data analysis was performed in an automated manner using pipelines described in the paper.

Reporting for specific materials, systems and methods

We require information from authors about some types of materials, experimental systems and methods used in many studies. Here, indicate whether each material, system or method listed is relevant to your study. If you are not sure if a list item applies to your research, read the appropriate section before selecting a response.

Materials & experimental systems

n/a	Involved in the study	
☐	☒	Antibodies
☐	☒	Eukaryotic cell lines
☒	☐	Palaeontology and archaeology
☒	☐	Animals and other organisms
☒	☐	Clinical data
☒	☐	Dual use research of concern
☒	☐	Plants

Methods

n/a	Involved in the study	
☒	☐	ChIP-seq
☒	☐	Flow cytometry
☒	☐	MRI-based neuroimaging

Antibodies

Antibodies used

For Western blot, we used the following antibodies: CHD7 (Thermo Fisher Scientific, PA5-72964), HSP90 (CST, 4874S), Sox10 (CST, 89356S), PCBP1 (sc-137249), ZNF114 (NBP1-81181), Actin (Abcam, ab179467), horseradish peroxidase-conjugated secondary antibodies (Abcam, ab6721 and ab6789). For immunofluorescence, we used the following antibodies: Geminin (Abcam, ab195047), Cas9 (Sigma, SAB4200701-25UL), Alexa Fluor 647-labeled secondary antibody (Thermo Fisher Scientific, A21237), Alexa Fluor 488-labeled secondary antibody (Invitrogen, A11034).

Validation

We relied on validations of the antibodies from manufacturers and published studies. CHD7 antibody has been tested on SH-SY5Y cell for immunofluorescence analysis and mouse intestine tissue for immunohistochemical analysis. HSP90 antibody has been tested on HeLa, NIH/3T3, C6 and COS cells for western blot analysis. Sox10 antibody has been tested on SK-MEL-5 cells. PCBP1 antibody has been tested on HeLa, NIH/3T3, K-562 and Sol8 cells. ZNF114 antibody has been tested on RT-4 and U-251 MG cells for western blot analysis. Actin antibody has been tested on mouse and rat tissue lysate for western blot analysis.

Eukaryotic cell lines

Policy information about cell lines and Sex and Gender in Research

Cell line source(s)

A549, human non-small cell lung cancer cell line, ATCC CCl-185.
hTERT-RPE1, hTERT-immortalized retinal pigment epithelial cell line, ATCC CRL-4000.
H1, human embryonic stem cell line, WiCell WA01.
HEK-293FT, human embryonic kidney cell line, used for lentivirus production, Thermo Fisher Scientific R70007.

Authentication

No authentication was conducted.

Mycoplasma contamination

A549 cells have tested negative for mycoplasma.

Commonly misidentified lines
(See ICLAC register)

No commonly misidentified cell line has been used in this study.
