# Supplementary figures and images for: Perturb-tracing enables high-content screening of multi-scale 3D genome regulators
Source: Nat Methods. 2025 Apr 10;22(5):950–61. doi: 10.1038/s41592-025-02652-z (PMC12074983; doi:10.1038/s41592-025-02652-z)

CHD7

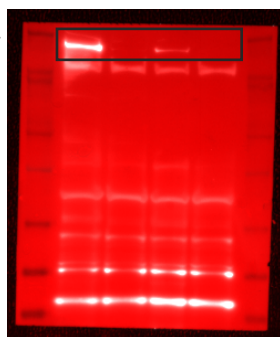

— 460 kDa

— 268 kDa

Actin B

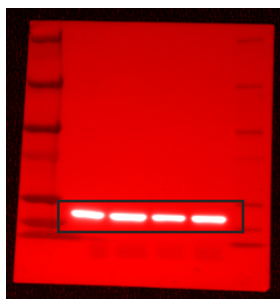

— 55 kDa

— 41 kDa

Supplement: Supplementary file 23 — Full image of a western blot (presented in Fig. 4a). [file 41592_2025_2652_MOESM23_ESM.pdf]

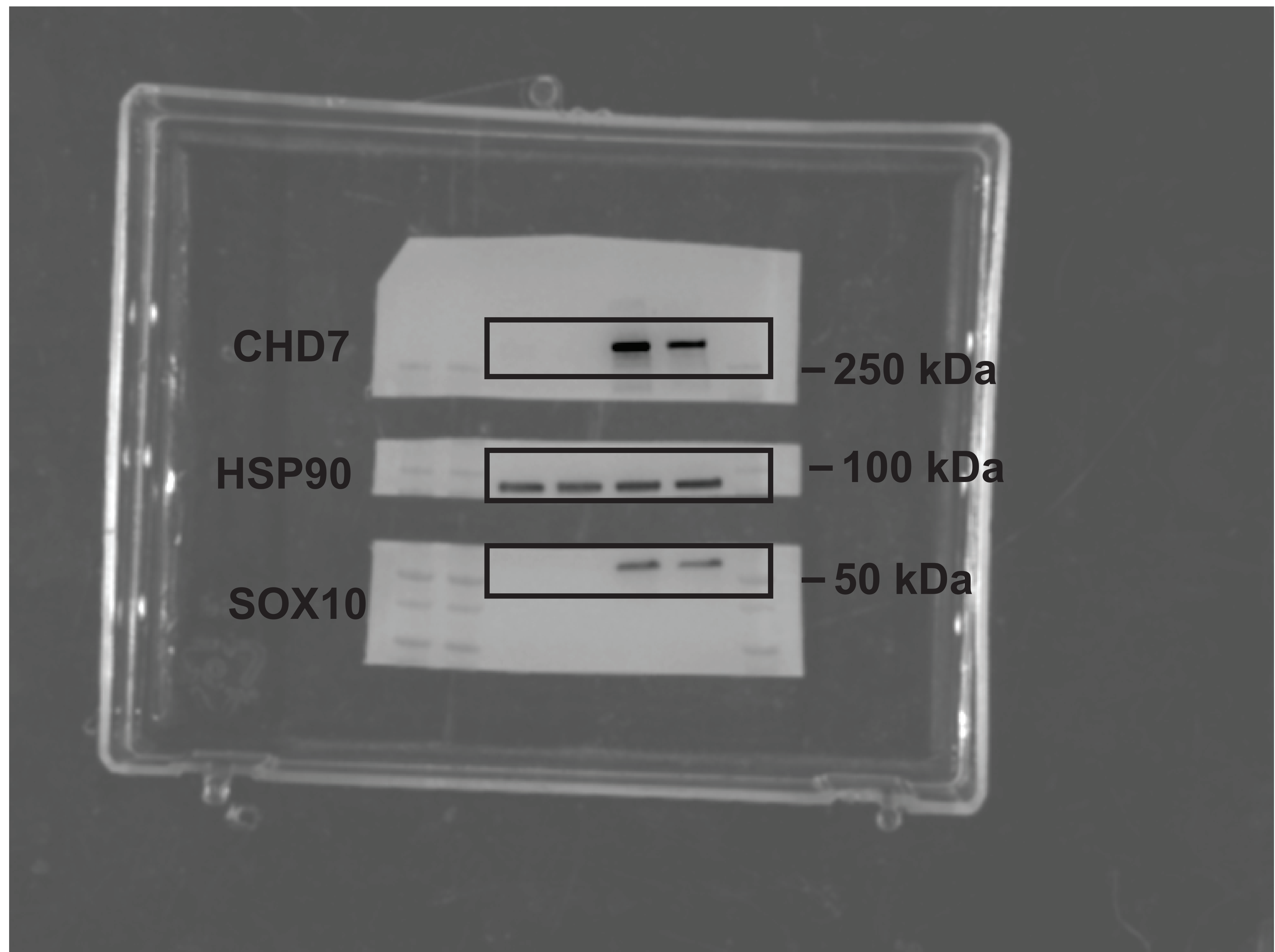

Supplement: Supplementary file 24 — Full image of a western blot (presented in Extended Data Fig. 5a). [file 41592_2025_2652_MOESM24_ESM.pdf]

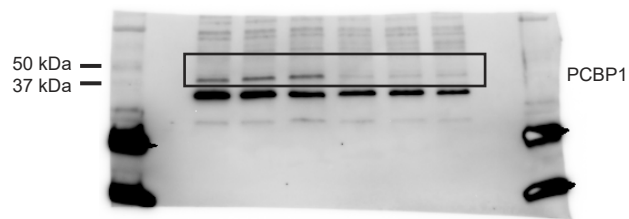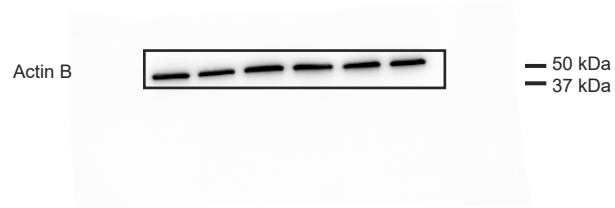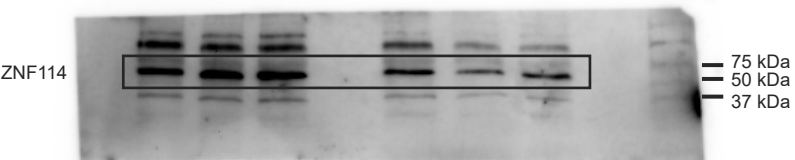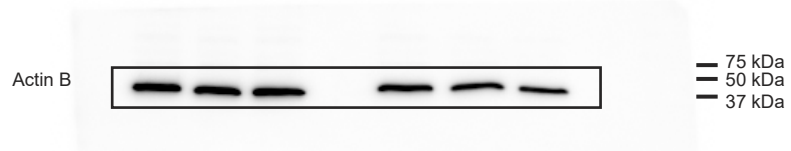

Supplement: Supplementary file 25 — Full image of western blots (presented in Extended Data Fig. 6a,b). [file 41592_2025_2652_MOESM25_ESM.pdf]
